# Paternal Prenatal and Lactation Exposure to a High-Calorie Diet Shapes Transgenerational Brain Macro- and Microstructure Defects, Impacting Anxiety-Like Behavior in Male Offspring Rats

**DOI:** 10.1523/ENEURO.0194-23.2023

**Published:** 2024-02-09

**Authors:** Luis A. Trujillo-Villarreal, Gabriela Cruz-Carrillo, Diego Angeles-Valdez, Eduardo A. Garza-Villarreal, Alberto Camacho-Morales

**Affiliations:** ^1^Department of Biochemistry, College of Medicine, Universidad Autónoma de Nuevo León, San Nicolás de los Garza, Nuevo Leon 64460, Mexico; ^2^Neurometabolism Unit, Center for Research and Development in Health Sciences, Universidad Autónoma de Nuevo León, San Nicolás de los Garza, Nuevo Leon 64460, Mexico; ^3^Instituto de Neurobiología, Universidad Nacional Autónoma de México, Campus Juriquilla, Queretaro 76230, Mexico

**Keywords:** anxiety, maternal programming, neuroimaging, transgenerational inheritance

## Abstract

Prenatal exposure to high-energy diets (HED) increases the susceptibility to behavioral alterations in the male offspring. We addressed whether prenatal HED primes the transgenerational inheritance of structural brain changes impacting anxiety/depression-like behavior in the offspring. For this, we used female Wistar rats exposed to a HED [cafeteria (CAF) diet, *n* = 6] or chow [control (CON) *n* = 6] during development. Anxiety and depression-like behavior were evaluated in filial 1 (F1), filial 2 (F2), and filial 3 (F3) male offspring using the open field (OFT), elevated plus maze, novelty suppressed feeding (NSFT), tail suspension (TST), and forced swimming tests. Structural brain changes were identified by deformation-based morphometry (DBM) and diffusion tensor imaging using ex vivo MRI. We found that the F1, F2, and F3 offspring exposed to CAF diet displayed higher anxious scores including longer feeding latency during the NSFT, and in the closed arms, only F1 offspring showed longer stay on edges during the OFT versus control offspring. DBM analysis revealed that CAF offspring exhibited altered volume in the cerebellum, hypothalamus, amygdala, and hippocampus preserved up to the F3 generation of anxious individuals. Also, F3 CAF anxious exhibited greater fractional anisotropy and axial diffusivity (AD) in the amygdala, greater apparent diffusion coefficient in the corpus callosum, and greater AD in the hippocampus with respect to the control. Our results suggest that prenatal and lactation exposure to HED programs the transgenerational inheritance of structural brain changes related to anxiety-like behavior in the male offspring.

## Significance Statement

Prenatal and lactation programming by HED exposure primes the transgenerational inheritance of aberrant behaviors in the offspring. A deeper understanding of the effect of diet on structural brain alterations that code for anxiety-like behavior and their transgenerational inheritance may allow the identification of new targets for future interventions. We used MRI to globally and selectively characterize diet-dependent structural brain changes and anxiety-like behavior in the F1, F2, and F3 generations of rats. The current findings suggest that prenatal and lactation exposure to HED affects brain structure leading to anxiety-like behavior, which is transgenerational preserved in the F2 and F3 generations.

## Introduction

Anxiety and depressive disorders are characterized by symptoms of restlessness, fatigue, irritability, sadness, loss of interest or pleasure, feelings of guilt or low self-worth, and poor concentration in humans (American Psychiatric Association, 2013). Epidemiological studies indicate that 3.6% of the global population experiences anxiety disorders, while estimates from 2015 suggest that as much as 4.08% of people live with depression (World Health Organization, 2017). Notably, the COVID-19 pandemic promoted a dramatic increase in anxiety and depression diagnoses, adding up to 13.7 and 9.6% of total worldwide cases, respectively, regardless of sex, age, race, or geographic location (Deng et al., 2021; Sahebi et al., 2021; Schafer et al., 2022).

The causes of depression and anxiety are largely unknown. It is hypothesized that prenatal factors and postnatal lifestyle factors together with genetic predisposition may increase the susceptibility to present these disorders. Prenatal exposure to a high-energy diet (HED) has been shown to induce depression-like behavior in the offspring of rats (Peleg-Raibstein et al., 2012; Sasaki et al., 2014; Winther et al., 2018; A. L. de la Garza et al., 2019; Trujillo-Villarreal et al., 2021). Prenatal programming refers to the process by which a fetus is exposed to healthy or adverse insults during critical periods of development, setting permanent changes in metabolism, immunity, and mood-related disorders after birth (Marques et al., 2015; Khambadkone et al., 2020). However, the extent to which prenatal programming contributes to the development of depression/anxiety diagnoses in the offspring and their transgenerational effect is still far from being understood. Animal studies are trying to establish a causal link between prenatal conditions and the transgenerational inheritance of depression/anxiety behavior in the offspring. For example, chronic and unpredictable maternal separation in early life induces depressive-like behaviors in F1, F2, and F3 generations, which correlates with altered methylation in the MeCP2, CB1, and CRFR2 genes in the brain (Franklin et al., 2010). Mothers receiving systemic administration of the viral mimetic polyinosinic:polycytidylic acid [poly(I:C)] also prime the intergenerational inheritance of depression-like behavior in the F2 offspring (Ronovsky et al., 2017). This evidence suggests the effect of prenatal exposure to nutritional or immune stimuli that prime the inter- and transgenerational inheritance of anxiety and depression-like behavior in the offspring.

The idea that maternal diet can impact the phenotype of subsequent generations is not new (Aiken et al., 2016). A growing body of evidence now suggests that the male environment and feeding patterns may also impact the progeny (McCoy et al., 2018, Soubry, 2018, de Lauzon-Guillain et al., 2022). The primary goal of our study was to determine whether the progeny of males exposed to a high-calorie diet during the prenatal period possessed the potential to inherit behaviors associated with anxiety for several generations. While it is established that offspring of over nourished males exhibit such effects, our focus was to ascertain whether prenatal exposure alone could program them to elicit a similar impact.

Offspring's vulnerability to anxiety and depression-like behavior may be attributed to changes in the structure of the brain. Both preclinical and clinical investigations support the notion that abnormal structure and function in several brain regions, including the amygdala, periaqueductal gray, dorsomedial prefrontal cortex, bed nucleus of the stria terminalis, paraventricular nucleus of the hypothalamus, and the locus ceruleus, potentially play a role in the development of anxiety and depressive disorders (Cross et al., 2016; Garcia, 2017; Donnici et al., 2021; Tung et al., 2021); however, the cause of such brain volume alterations in subjects diagnosed with anxiety and depression-like behavior has not been completely identified. A recent study found that fetal programming through exposure to a high-energy diet (HED) led to reduced volume in the nucleus accumbens, hippocampus, and prefrontal cortex of rats displaying depression-like behavior (Trujillo-Villarreal et al., 2021). Although there is a detrimental effect of prenatal exposure to HED, which alters brain structures related to behavior in the first generation of offspring, there is no evidence in preclinical or clinical studies of the possible effect of prenatal diet on the transgenerational inheritance of anxiety and depression-like behavior in the offspring.

In our previously published work, our group helped establish that maternal exposure to a cafeteria diet leads to increased susceptibility to depression-like behavior in rats (Trujillo-Villarreal et al., 2021). However, the novel aspect of the present work lies in its examination of the transgenerational implications of this phenomenon. Unlike our prior investigation, where the focus was on the direct maternal effect, this study delves into the inheritance and persistence of these vulnerabilities across multiple generations. By extending our research to encompass transgenerational effects, we looked for fresh insights into the long-lasting and potentially heritable consequences of maternal diet on behavioral susceptibility in the offspring. This approach underscores the dynamic interplay between environmental factors and heredity, broadening our understanding of the scope and implications of maternal diet-induced anxiety susceptibility.

Here, we seek to address the effect of prenatal and during lactation exposure to HED on structural brain alterations in the brain of F1 offspring with anxiety and depression-like behavior and their potential transgenerational inheritance to the F2 and F3 generations.

## Materials and Methods

### Animals and housing

Programing and mating experiments were performed using 12-week-old males and 10-week-old virgin female Wistar rats. Animals were handled according to the NOM-062-ZOO-1999 for the care and use of laboratory animals, with the approval of the Universidad Autónoma de Nuevo León Animal Care Committee (BI0002). Rats were housed in a room with a 12 h light/dark cycle (7:00–19:00). Behavioral tests were conducted during the day phase of the rat's diurnal cycle. Food and water were available ad libitum.

### Prenatal programming model by cafeteria (CAF) diet exposure

**Figure 1.**
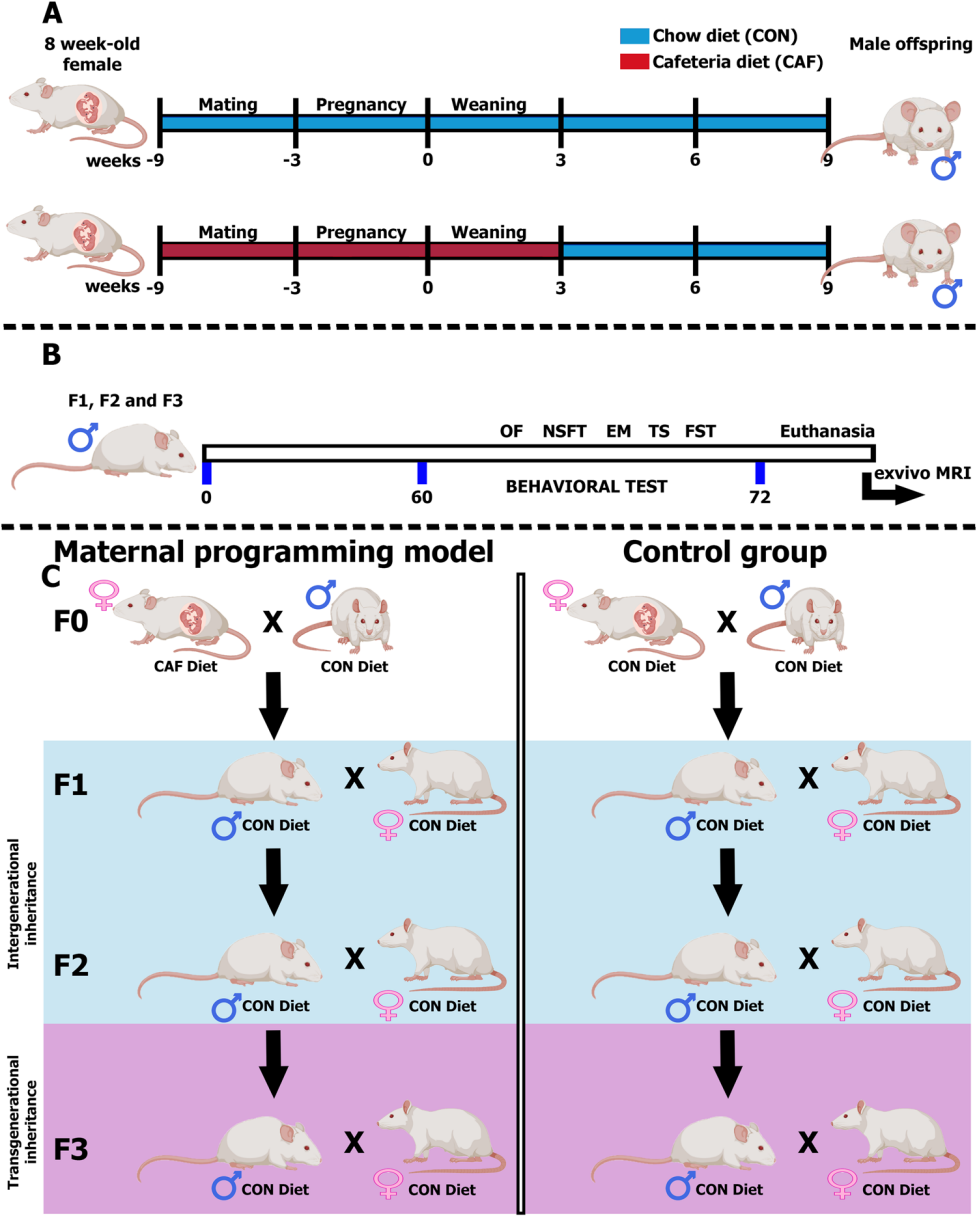
Experimental design of the fetal programming model. ***A***, Fetal programming model. Female rats were randomized and were exposed to two dietary formulas: Control chow or CAF diet for a total of 9 weeks, including 3 weeks before pregnancy, 3 weeks during pregnancy, and until weaning. Rats were mated with 12- to14-weeks-old Wistar males, 300–350 g. After weaning offspring was swift to control chow diet to follow the behavioral tests. ***B***, Timeline of behavioral tests used in every male offspring F1, F2, and F3. CAF, cafeteria diet; CON, chow diet; OF, open field test; NSFT, novelty suppressed feeding test; LDB, light–dark box test; EM, elevated maze. ***C***, Graphical representation of the maternal programming model design to study the transgenerational effect of maternal diet illustrating the intergenerational inherence (F1–F2) and transgenerational inherence (F1–F3).

A cohort of inbred 10-week-old, 200–250 g female rats (*n* = 12) was initially housed collectively for a minimum of 10 d to facilitate acclimatization. Rats were unrestricted access to a standard chow diet and water. Subsequently, the rats were allocated into two distinct dietary groups: control chow (CON; *n* = 6) and cafeteria (CAF) diet (*n* = 6; Camacho et al., 2017; Montalvo-Martínez et al., 2018; A. L. de la Garza et al., 2019; Trujillo-Villarreal et al., 2021; Camacho-Morales et al., 2022). Diet formulation for CON diet contained a caloric density of 3.35 kcal/g divided into 58% carbohydrates, 13% lipids, and 29% proteins (Purina LabDiet, cat. #5001). CAF diet was made of liquid chocolate, cookies, bacon, fried potatoes, standard diet, and pork pâté at a 1:1:1:1:1:1:2 ratio. CAF chow has a density of 3.72 kcal/g total divided into 39% carbohydrates, 49% lipids, 12% proteins, and 513.53 mg of sodium. The females were fed CON or CAF diet for 9 weeks, including 3 weeks of prepregnancy, pregnancy, and lactation. At the beginning of Week 4, female rats were mated with Wistar males (12–14 weeks old, 300–350 g, one male/three females) for 2 d. A possible mating day was registered by the observation of a vaginal plug. Male offspring from mothers exposed to CON (*n* = 11) or CAF (*n* = 20) diet were weaned by postnatal day 21. Offspring from mothers exposed to CAF diet were switched to CON diet after weaning (Fig. 1*A*).

### Behavioral phenotyping for anxiety-like and depression-like behavior

Behavioral phenotyping in the F1 offspring was performed in males at 8 weeks of age. Female offspring from mothers exposed to CON or CAF diets were weaned by postnatal day 21 and allocated to a second experimental protocol (unpublished data). Behavioral phenotyping for anxiety-like behavior was carried out by using the open field (OF) test, novelty suppressed feeding test (NSFT), and elevated plus maze (EM) test. For depression-like behavior, we used tail suspension (TST) and forced swimming test (FST; Fig. 1*B*).

#### OF

We used an open field arena made of white acrylic walls and a black floor (50 × 50 × 50 cm) illuminated with 300 lux. The rats were gently held by their tail base and positioned within one corner of the open field arena. Subjects were allowed to explore the apparatus for 10 min. After the 10 min test, rats were returned to their home cages, and the open field area was cleaned using 70% ethyl alcohol. The activity was video recorded for 10 min using an automatic motion sensor system (Omnialva). In order to verify locomotor activity, we quantified the total distance traveled and percentage of inactivity. We evaluated the time spent in the center and on the edges of the arena as an anxiety parameter using ToxTrac software (Rodriguez et al., 2018).

#### NSFT

Rats were subjected to an 18 h food deprivation period. Subsequently, a single chow diet pellet was placed in the center of the open field arena, which measured 50 × 50 × 50 cm illuminated with 300 lux. The test commenced by positioning the rats in one of the corners of the arena, and the time taken to reach the pellet (latency to feed) was recorded. This test is based on the assumption that exposure to a novel environment induces anxiety, leading to delayed food intake (Ramaker and Dulawa, 2017). Rats that successfully reached the pellet were individually housed and provided with pre-weighed chow food pellets. To mitigate the confounding effect of hunger levels during the behavioral test, we quantified the amount of food ingested after a 10 min period in their home cage, where no stressors were present.

#### EM

Our maze features a white acrylic surface and comprises four arms, each measuring 50 cm in length. Two of these arms are open, lacking walls, while the remaining two are enclosed by walls that stand at a height of 30 cm. Notably, the open arms were illuminated with a brightness of 300 lux. Each arm of the maze is attached to sturdy plastic legs, such that it is elevated 50 cm off its base. Subjects were placed in the middle of the maze and allowed to explore the arms for 5 min. We quantified the time spent in open and closed arms.

#### TS

Depression-like behavior was examined using the tail suspension test. The election of the TST was based on several benefits over the FST. These include not being influenced by variations in water temperature during swimming, fewer errors in rats exhibiting dyskinesia, greater responsiveness to a broader spectrum of antidepressant substances, and no hypothermia induced (Castagné et al., 2010). A previous protocol reported for the TST (Joksimovic et al., 2017) was modified and followed. The test apparatus consisted of white acrylic walls, with one open wall through which the animals could be video recorded and illuminated. Each rat was suspended by the tail 60 cm above the floor of the chamber and *hung from a ring of tape coated with cotton 1 cm from their tails to reduce discomfort*. The resultant behavior was recorded by a video camera for 6 min. The behavior was later analyzed to determine the total duration of immobility. Data acquisition was measured manually.

#### FST

The FST was also used to examine depression-like behavior. The apparatus consisted of three Plexiglas cylinders [60 cm (height) × 30 cm (diameter)]. Each cylinder was placed surrounded by white curtains to avoid rat-to-rat visual contact and with one wall open through which the behavior was recorded. The cylinders were filled with water (23°C) to a depth of 70 cm. The rat was placed in the cylinders for 7 min and video recorded after 2 min of swim conditioning. The behavior of the rat was analyzed to determine the total duration of immobility. In this test, immobility was defined as the period when the animals stopped struggling for ≥1 s. Data acquisition was measured manually.

### Transgenerational inheritance of anxiety-like and depression-like behavior in the offspring

Male Wistar rats of the F1 and F2 generation, with anxiety-like and depression-like behavior, were mated with virgin females (*n* = 12; 200–250 g, 10 weeks old) to generate the F2 and the F3 offspring, respectively. Briefly, three F1 males with the highest score for anxiety-like and depression-like behavior phenotype and born from CON- or CAF-fed dams were mated with virgin control females (*n* = 2 females per male, *n* = 6 virgin females) for 2 d. After mating, males were removed from the home cage (Fig. 1*C*). The presence of a vaginal plug denoted gestation day 1. The pregnant rat was individually housed and fed with a chow diet during pregnancy and lactation. Accordingly, F2 male offspring from CON- (*n* = 29) or CAF-fed dams (*n* = 22) were weaned by day 21 postpartum and maintained with a chow diet until reaching 8 weeks of age to perform behavioral phenotyping as described for the F1 offspring (Fig. 1*B*). To generate the F3 cohort, we selected three F2 males born from CON- or CAF-fed dams displaying the major anxiety-like behavioral phenotype based on classification through PCA analysis (refer to the PCA section for details). Females were mated with virgin control females (*n* = 2 females/male, *n* = 6 virgin females) for 2 d. The F3 offspring (CON, *n* = 28; CAF, *n* = 42) were weaned by day 21 postpartum and maintained with a chow diet until reaching 8 weeks of age to perform behavioral phenotyping as described for the F1 offspring (Fig. 1*B*). The F1, F2, and F3 female offspring was allocated to a second experimental protocol that is currently under investigation.

### PCA

The PCA was performed using the FactoMineR package (Lê et al., 2008) in “R” to classify behavioral outcomes dictated by prenatal diet exposure in the F1, F2, and F3 offspring (CON vs CAF diet). The purpose of PCA is to reduce dimensionality by deriving a small number of linear combinations (principal components) from a set of variables from the behavioral test used to measure anxiety and depression-like behavior (latency to feed in the open field, latency to feed in homecage, fecal boli in the NSFT, index in FST and EM) that retain as much of the information in the original variables as possible (Jang, 2021). Once the data has been transformed into a lower-dimensional space by PCA, k-means clustering is applied using the ClusterR package (Mouselimis, 2022). K-means is an unsupervised clustering algorithm that groups data points (in this case, rats) into clusters based on their similarity in the reduced feature space. This procedure performs a disjoint cluster analysis based on the distances computed from one or more quantitative variables, using Euclidean distances (k-means model) to calculate cluster centers. Overall, this process combines dimensionality reduction with clustering techniques to classify rats based on their behavioral variables, providing a structured approach to understanding and categorizing behavioral patterns in the rat population under study.

### Ex vivo fixation and MRI

After behavioral phenotyping, the F1, F2 and F3 rats at postnatal day 72 were intraperitoneally anesthetized with an overdose of 1 ml of pentobarbital (PiSA Agropecuaria) and transcardially perfused following standardized methods as reported (Trujillo-Villarreal et al., 2021; Cárdenas-Tueme et al., 2022; Maldonado-Ruiz et al., 2022). For MRI acquisition, the skulls were submerged and fixed inside plastic tubes filled with Fomblin (Sigma 317950-100G), and imaging was performed in a 16-cm-bore 7T Bruker PharmaScan MRI scanner using a 2 × 2 array surface rat head coil, interfaced to a Paravision 6.0.1 and 7.0.1 console (Bruker) with an inner diameter of 72 mm. We scanned a T1w sequence and a diffusion-weighted sequence [diffusion-weighted imaging (DWI)]. The T1w had a isometric voxel resolution 0.060 mm 3D FLASH, TR/TE = 39.26/11.00 ms, flip angle = 20°, averages = 1, matrix = 364 × 500 × 336, pixel bandwidth = 55.556 Hz, FOV = 22 × 30 × 20 mm, and number of slices = 376. The DWI was acquired using an echo-planar imaging sequence with the following parameters: TR = 2,000 ms, TE = 22.85 ms, slice thickness = 195 μm, FOV 22 × 15 mm, matrix = 126 × 86, and scan time = 12 min. DWI were acquired in a multishell acquisition protocol with *b* = 650 s/mm^2^, *b* = 1,250 s/mm^2^, and *b* = 2,000 s/mm^2^ and 60 directions. Morphological analysis was performed by converting DICOM to MINC format and then preprocessed using an in-house pipeline based on MINC-Tools and the pydpiper pipeline (https://github.com/Mouse-Imaging-Centre/pydpiper). All analyses were performed using pydpiper (Friedel et al., 2014), R statistics (R Core Team, 2013), R (RStudio Team, 2015), and the RMINC (Lerch et al., 2017) and tidyverse (Wickham, 2017) packages.

### MRI image quality control

Quality control was performed on every MRI image obtained from each subject at each step of the process in this study.

#### Image acquisition

During the image acquisition phase, we classified the images on a scale from 1 to 3 based on their quality. A rating of 1 indicated images that successfully achieved exceptional clarity and sharpness, high signal-to-noise ratio with minimal noise, motion, or artifacts, and displayed distinct and well-defined anatomical structures.

#### Image preprocessing

In the image preprocessing stage, we ensured that all images were properly registered and aligned to a common anatomical template. Additionally, we carefully evaluated each image for the presence of any evident structural abnormalities or artifacts that could potentially impact the subsequent analysis.

By implementing this rigorous quality control process, we aimed to ensure the reliability and accuracy of the MRI data used in our study. The systematic assessment of image quality allowed us to identify and address any issues or limitations that could affect the interpretation of the results.

### Deformation-based morphometry analysis

After performing quality control on the images, we selected a total of 109 scans out of the initial 152 scans for further analysis. These 109 scans were derived from groups F1 (14 scans), F2 (42 scans), and F3 (53 scans), as they successfully passed the quality control assessment. All images were converted from DICOM to MINC format and then preprocessed using an in-house pipeline based on MINC-Tools and ANTs, which performed the following steps: center image, full mask, and N4 bias field correction. We then used an image registration-based approach to assess anatomical differences between groups. Image registration finds a smooth spatial transformation that best aligns one image to another such that corresponding anatomical features are superimposed. We used an automated intensity-based, group-wise registration approach to align all brains, into a common coordinate system by generation (F1, F2, and F3), yielding an average image of the T1w scans per generation. The deformation that aligns the images becomes a summary of their differences. To assess volume differences between groups (CAF vs CON) within each generation (F1, F2, and F3), we performed a deformation-based morphometry (DBM) analysis. Deformations were then mapped from the individual scans back to the average image. The final deformation fields were computed with a greedy symmetric diffeomorphic registration (the SyN algorithm in ANTs; Avants et al., 2008) and then inverted and blurred with a 0.2 mm FWHM Gaussian smoothing kernel. The relatives Jacobians determinants of these deformations were extracted, giving a measure of local volume expansion/contraction at every voxel in the brain (Chung et al., 2001). Relative Jacobian determinants explicitly model only the nonlinear part of the deformations and remove residual global linear transformations (attributable to differences in total brain size); hence, we focused on local volume (Lerch et al., 2017). These were used to assess differences between groups because they better estimate a normal distribution (Leow et al., 2007). We localized brain areas using the Fisher 344 atlas (Goerzen et al., 2020). Details about the pipeline commands are available as open access (https://github.com/LuisTrujillo11/Programmed_Anxiety_model).

### DWI analysis

The DWI data sets were denoised and corrected for motion and eddy-current–induced distortions using linear transformations (12 degrees of freedom). The Mrtrix 3.0 software package was used to estimate the diffusion tensor (Tournier et al., 2019; Luna-Munguia et al., 2021), from which we obtained the corresponding eigenvalues (*λ*1, *λ*2, and *λ*3). From these, we created quantitative maps of fractional anisotropy (FA), apparent diffusion coefficient (ADC), axial diffusivity (AD, i.e., *λ*1; *D*||), and radial diffusivity ([*λ*2 + *λ*3]/2; *D*┴; RD). Diffusion tensor imaging (DTI) parameters were analyzed using the main diffusivity maps (aided by the nondiffusion-weighted images) to manually outline regions of interest (ROIs) based on the Paxinos atlas version 6 (Paxinos and Watson, 2013) corpus callosum (CC), fornix, fimbria, internal capsule, cerebellar lobule 3, cerebellar lobule 6, hippocampus, and amygdala for each F1, F2, or F3 offspring.

### Statistical analysis

All behavioral statistical analyses, including testing the normality of data distribution and two-way ANOVA post hoc Bonferroni’s and Student's *t* tests of behavioral parameters, were performed using GraphPad Prism 8.0.1 software, and a corrected *p*-value <0.05 was considered significant. All results were tested for normality using the Shapiro–Wilk test and effect size using Cohen's *d*. The statistical analysis on DBM was done using R statistics (R Core Team, 2013) and the RMINC package (https://github.com/Mouse-Imaging-Centre/RMINC), with the relative Jacobian (volume) determinants as the dependent variable in a general linear model, “prenatal programming group” as the independent variable (between subjects), and “phenotype” as a covariate. Additionally, we included the variable “body weight” as a covariate in the general linear model due to its impact on brain volume, as it showed significant differences among the groups (Extended Data Fig. 5-1). We compared the groups using a general linear model, and data were corrected for multiple comparisons using the false discovery rate (FDR) at 10%. Furthermore, we extracted the *t* values from significant peaks and we evaluated the interaction of prenatal diet and the phenotype on local brain volume changes using the volume value calculated for the F1, F2, and F3 generations followed by a post hoc Bonferroni’s test to compare each Jacobian value from significant peaks for all groups. We compared the mean FA, AD, ADC, and radial diffusion (RD) values from each ROI for groups per generation using ANOVA post hoc Bonferroni’s test. Data are presented as mean ± SEM for all data. Details about the statistical analysis are available in the open-access R script (see above).

### Data availability

The spike train and behavioral data that support our findings are available at: https://github.com/LuisTrujillo11/Programmed_Anxiety_model).

## Results

### Prenatal exposure to HED primes the transgenerational inheritance of anxiety-like behavior in the offspring

**Figure 2.**
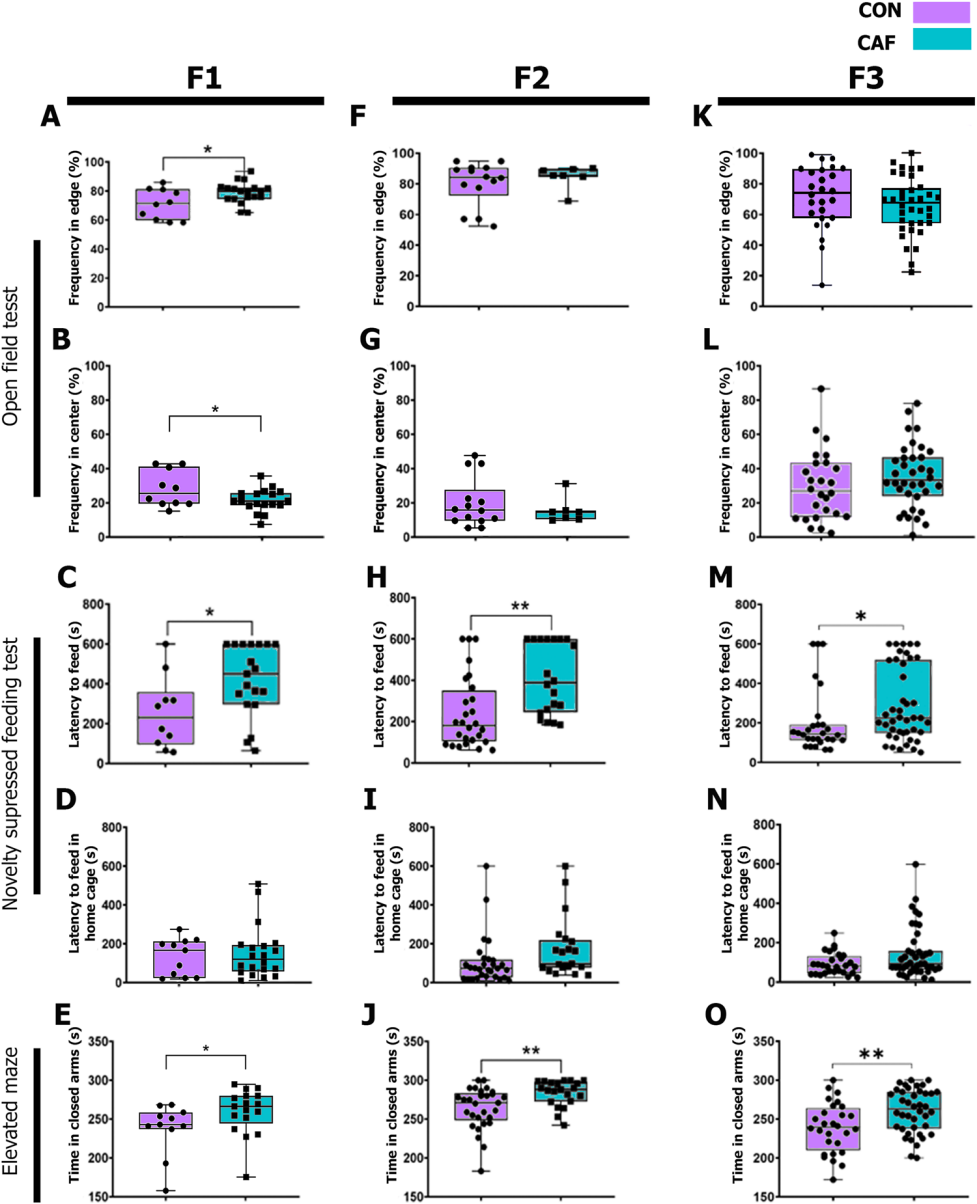
Effect of maternal programming in anxiety-like behavior in inter- and transgenerational inheritance. Frequency in edges was measured during open field test and compared the CAF group from (***A***) F1, (***F***) F2, and (***K***) F3 generations with their respective CON group. The results are expressed as mean ± SEM, followed by ANOVA post hoc Tukey. *p < 0.05 versus control group. Frequency in the center was measured during open field test and compared the CAF group from (***B***) F1, (***G***) F2, and (***L***) F3 generations with their respective control group. The results are expressed as mean ± SEM, followed by ANOVA post hoc Tukey. *p < 0.05 versus the control. Latency to feed was determined by comparing the time required to reach the food at the center of the arena in the CAF group from (***C***) F1, (***H***) F2, and (***M***) F3 generations compared with their respective control group. The results are expressed as mean ± SEM, followed by ANOVA post hoc Tukey. *p < 0.05, **p < 0.01. Quantification of food intake in cage after fasting in the CAF from (***D***) F1, (***I***) F2, and (***N***) F3 generations compared with their respective CON group. The results are expressed as mean ± SEM, followed by ANOVA post hoc Tukey *p < 0.05. Comparison of time in closed arms during elevated maze was analyzed between the CAF and CON groups from (***E***) F1, (***J***) F2, and (***O***) F3 generations. The results are expressed as mean ± SEM, followed by ANOVA post hoc Tukey *p < 0.05 and **p < 0.01. CAF, cafeteria diet group; CON, chow diet group. For further behavioral parameters, please refer to Extended Data Figure 2-1.

We first evaluated anxiety-like behavior using an open field test in the F1 male subjects prenatally and during lactation exposed to CON or CAF diets and its behavioral phenotype preservation in the F2 and F3 generations. No significant interaction effect was observed in the ANOVA analysis of the behavioral tests assessing anxiety (Extended Data Table 2-1). We evaluated the effect of anxiety-related behaviors in the offspring (F1, F2, and F3) generations. Analyzing the offspring groups individually allows us to discern any distinctive effects of the filial generation on anxiety-related behaviors and explore potential intergenerational changes. We found that prenatal and lactation exposure to CAF diet primes anxiety-like behavior in F1 rats, evidenced by longer time spent in the edges (Fig. 2*A*; F1 CON *M* = 71.14 SD = 10.44 vs F1 CAF *M* = 78.90 SD = 7.26, *t*_(27)_ = 2.35 *p = 0.0264, *d* = 0.741) and shorter time spent in the center of the arena during the open field test when compared with the F1 rats exposed to CON diet (Fig. 2*B*; F1 CON *M* = 27.14 SD = 10.60 vs F1 CAF *M* = 20.33 SD = 6.64, *t*_(27)_ = 2.095 *p = 0.0461, *d* = −0.640).

10.1523/ENEURO.0194-23.2023.t2-1 Table 2-1 Results of Two-Way Analysis of Variance (ANOVA) for diet group and filial group comparison in behavioral variables. Download Table 2-1, DOCX file.

10.1523/ENEURO.0194-23.2023.f2-1 Figure 2-1 Box plots comparing food intake in the home-cage after reaching the pellet in the Novelty Suppressed Feeding Test (NSFT) and time spent in open arms during the Elevated Maze (EM) across groups categorized by F0 diet (CON, CAF) and offspring. Panels A and D show F1, panels B and E show F2, and panels C and F show F3. Significant differences between groups are denoted by asterisks: * p < 0.05, ** p < 0.01, and *** p < 0.001. Specific group differences were determined through post hoc t-test analysis. Download Figure 2-1, TIF file.

Next, we examined hypophagia induced by exposing subjects to a novel environment during the NSFT. We found that F1 rats exposed to CAF diet experienced more latency to reach the food pellet in the center of the arena (Fig. 2*C*; F1 CON *M* = 254.2 SD = 181.7 vs F1 CAF *M* = 420.5 SD = 181.6, *t*_(27)_ = 2.34 *p = 0.0267, *d* = 0.924), whereas no differences were found in the quantity of hunger evidenced by latency to feed in the home cage when compared with the control subjects (Fig. 2*D*; F1 CON *M* = 134.5 SD = 94.79 vs F1 CAF *M* = 155 SD = 137.2, *t*_(27)_ = 0.4376 *p* = 0.6649, *d* = 0.145) and amount of food intake (Extended Data Fig. 2-1A; F1 CON *M* = 1.9609 SD = 1.0193 vs F1 CAF *M* = 2.729 SD = 1.3414, *t*_(27)_ = −1.7885 *p* = 0.0854, *d* = −0.62). Finally, anxiety classification in the F1 rats exposed to CAF diet was confirmed during the EM, showing a significantly longer time spent in closed arms compared with the F1 control subjects (Fig. 2*E*; F1 CON *M* = 236.4 SD = 34.07 vs F1 CAF *M* = 264.5 SD = 21.23, *t*_(27)_ = 2.65 *p = 0.0137, *d* = 0.787), and no significance difference in time spent in open arms (Extended Data Fig. 2-1D; F1 CON *M* = 25.8 SD = 19.6796 vs F1 CAF *M* = 19.47 SD = 24.4594, *t*_(27)_ = 0.7549 *p* = 0.4582, *d* = 0.28). This evidence confirms that exposure to CAF diet during prenatal and lactation stages primes anxiety in F1 rats after birth.

It has previously been demonstrated that prenatal exposure to a high-energy diet (HED) establishes the transgenerational transmission of addiction-like behavior in the F2 and F3 progeny (Sarker et al., 2018; Montalvo-Martínez et al., 2022). We tested whether prenatally and during lactation exposing the F1 rats to CAF diet promotes the transgenerational inheritance of anxiety-like behavior in the F2 and F3 generations. The F2 and F3 offspring born from the F1 offspring with anxiety-like behavior did not show changes in the time spent on the edges (Fig. 2*F*; F2 CON *M* = 80.14 SD = 14.36 vs F2 CAF *M* = 84.70 SD = 7.36, *t*_(19)_ = 0.783 *p* = 0.4433, *d* = 0.316; Fig. 2*K*; F3 CON *M* = 70.33 SD = 20.35 vs F3 CAF *M* = 64.52 SD = 18.55, *t*_(60)_ = 1.167 *p* = 0.2479, *d* = −0.280) nor in the center of the arena during the open field test compared with the control subjects (Fig. 2*G*; F2 CON *M* = 19.86 SD = 14.36 vs F2 CAF *M* = 15.30 SD = 7.36, *t*_(19)_ = 0.783 *p* = 0.4433, *d* = −0.793; Fig. 2*L*; F3 CON *M* = 29.67 SD = 20.35 vs F3 CAF *M* = 35.48 SD = 18.55, *t*_(60)_ = 1.167 *p* = 0.2479, *d* = 0.318).

Notably, the F2 and F3 rats born from the F1 anxious subjects preserved anxiety traits including longer time spent to reach the pellet during the NSFT (Fig. 2*H*; F2 CON *M* = 202.7 SD = 125.3 vs F2 CAF *M* = 364.5 SD = 179.2, *t*_(19)_ = 2.42 **p = 0.0257, *d* = 1.160; Fig. 2*M*; F3 CON *M* = 188.3 SD = 146.8 vs F3 CAF *M* = 287.9 SD = 187.6, *t*_(68)_ = 2.343 *p = 0.0221, *d* = 0.593), and no significant difference was found in the latency to feed in their home cages after the NSFT (Fig. 2*I*; F2 CON *M* = 121.9 SD = 166.1 vs F2 CAF *M* = 191.3 SD = 205.1, *U* = 43.50 *p* = 0.3008, *d* = 0.265; Fig. 2*N*; F3 CON *M* = 92.57 SD = 55.40 vs F3 CAF *M* = 144.2 SD = 127.5, *U* = 494 *p* = 0.2063, *d* = 0.548) and amount of food intake (Extended Data Fig. 2-1B; F2 CON *M* = 2.9931 SD = 0.6286 vs F2 CAF *M* = 2.9366 SD = 0.5066, *t*_(47)_ = 0.3509 *p* = 0.7272, *d* = 0.1; Extended Data Fig. 2-1C; F3 CON *M* = 3.7889 SD = 0.6595 vs F3 CAF *M* = 3.5051 SD = 0.5306, *t*_(49)_ = 1.8961 *p* = 0.0637, *d* = 0.48). Moreover, results from EM showed that F2 and F3 subjects spent more time in the closed arms when compared with the F2 and F3 rats exposed to control diets (Fig. 2*J*; F2 CON *M* = 264.1 SD = 26.76 vs F2 CAF *M* = 283.7 SD = 16.62, *t*_(48)_ = 2.970 **p = 0.0046, *d* = 0.739; Fig. 2*O*; F3 CON *M* = 238.9 SD = 32.08 vs F3 CAF *M* = 260.4 SD = 28.31, *t*_(66)_ = 2.911 **p = 0.0049, *d* = 0.636) and no significance differences in time spent in open arms (Extended Data Fig. 2-1E; F2 CON *M* = 9.0344 SD = 15.3191 vs F2 CAF *M* = 3.0476 SD = 6.0454, *t*_(38)_ = 1.9093 *p* = 0.0636, *d* = 0.49; Extended Data Fig. 2-1F; F3 CON *M* = 14.4642 SD = 19.311 vs F3 CAF *M* = 8.5853 SD = 16.98, *t*_(53)_ = 1.3032 *p* = 0.1981, *d* = 0.33). These results confirmed that prenatal and lactation exposure to CAF diet seems to promote the transgenerational inheritance of anxiety-like behavior in the offspring.

### Prenatal exposure to HED primes the intergenerational inheritance of depression-like behavior in the offspring

**Figure 3.**
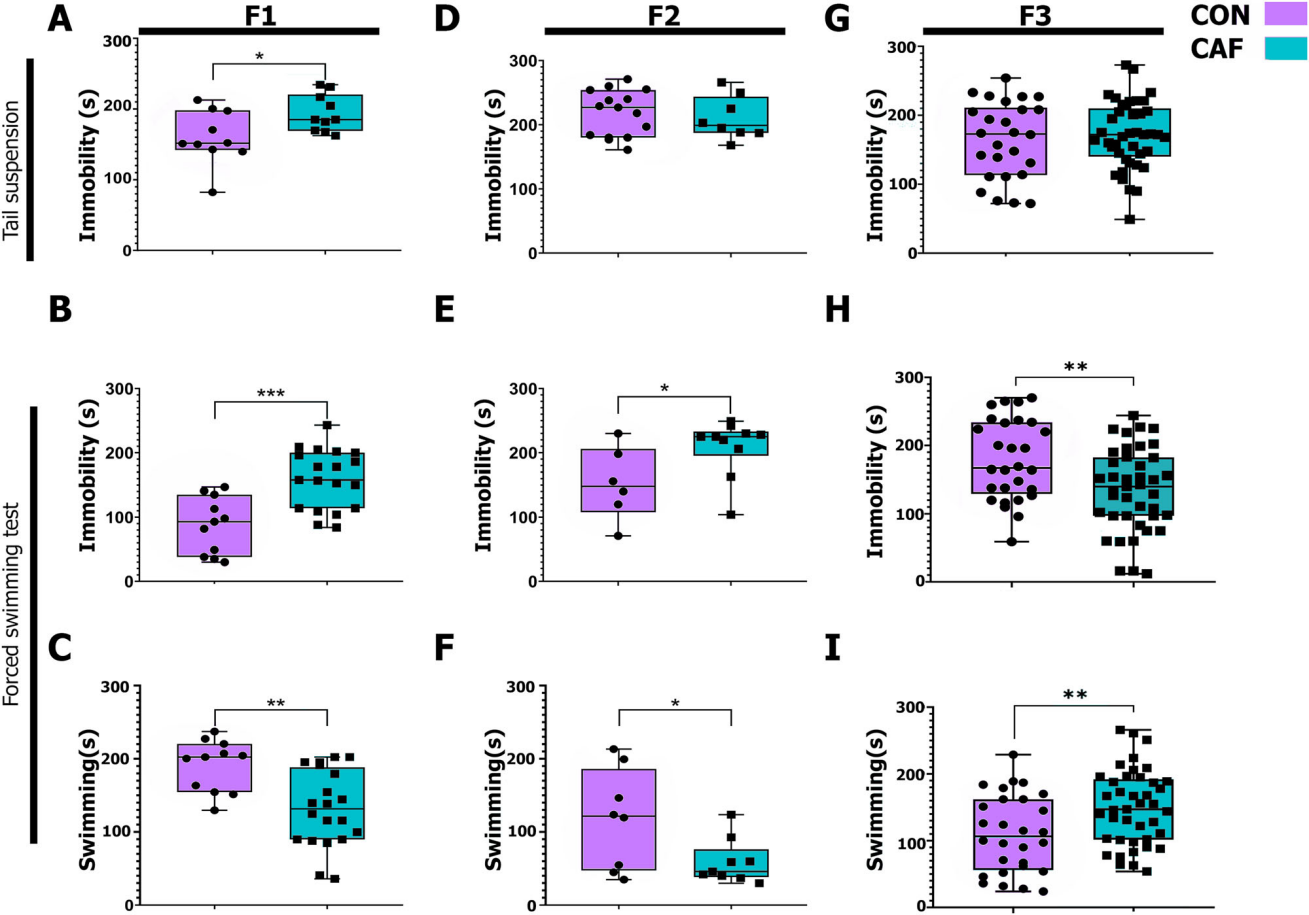
Effect of maternal programming in depression-like behavior in inter- and transgenerational inheritance. Seconds of immobility during tail suspension test were measured and compared CAF group from (***A***) F1, (***D***) F2, and (***G***) F3 generations with their respective CON group. The results are expressed as mean ± SEM, followed by ANOVA post hoc Tukey. *p < 0.05 versus control group. Seconds of immobility during FST were measured and compared CAF group from (***B***) F1, (***E***) F2, and (***H***) F3 generations with their respective CON group. The results are expressed as mean ± SEM, followed by ANOVA post hoc Tukey. *p < 0.05 versus control group. Contrarily, time spent swimming during FST was measured and compared CAF group from (***C***) F1, (***F***) F2, and (***I***) F3 generations with their respective CON group. Results are expressed as mean ± SEM, followed by ANOVA post hoc Tukey. *p < 0.05 versus control group. CAF, cafeteria diet group; CON, chow diet group.

Next, we evaluated immobility in the TST and FST to determine the effect of prenatal and lactation diet exposure on depression-like behavior in offspring. Immobility in TST and FST is commonly used to measure depression-related behavior (Mesripour et al., 2018; Seiffe et al., 2022). First, the data from the behavioral tests were analyzed using a two-way ANOVA, considering each behavioral parameter as the dependent variable and the diet consumed by the F0 and the offspring as the factors. Overall, a significant interaction effect was found only in immobility during FST (Extended Data Table 2-1, *p* = 0.00003). Post hoc Tukey's multiple comparisons revealed significant differences between CAF F2 and CON F1 (*p* = 0.0141), CON F3 and CAF F1 (*p* = 0.0039), CON F3 and CON F2 (*p* = 0.0169), CON F3 and CAF F2 (*p* = 0.00004), and CAF F3 and CAF F2 (*p* = 0.0032). However, since only an interaction effect was found, further comparisons were explored using independent *t* tests to analyze the effects of the factors separately. We found that prenatal and lactation exposure to CAF diet in the F1 offspring displayed higher immobility time during the tail suspension test (TST; *M* = 188.6, SD = 25.64) when compared with the control group (*M* = 155.6, SD = 36.86; Fig. 3*A*; *t*_(18)_ = 2.324, *p = 0.032, *d* = −0.897). Also, F1 rats prenatally and during lactation exposed to CAF diet showed higher immobility time (Fig. 3*B*; F1 CAF group *M* = 159.4, SD = 46.18; F1 CON group *M* = 87.36, SD = 44.13; *t*_(28)_ = 4.181, ***p = 0.0003, *d* = 1.539) and lower swimming time during the FST (*M* = 131.8, SD = 52.19) compared with the F1 subjects exposed to control diet (*M* = 191.4, SD = 35.32; Fig. 3*C*; *t*_(29)_ = 3.372, **p = 0.0021, *d* = 1.133). Accordingly, F2 and F3 rats born from the F1 CAF rats did not show significant alteration on the immobility time during the TST compared with the F2 (Fig. 3*D*; F2 CAF *M* = 210.3, SD = 33.83, F2 CON *M* = 218.1, SD = 35.60; *t*_(21)_ = 0.509 *p* = 0.6155, *d* = −0.219) and F3 rats exposed to CON diet (Fig. 3*G*; F3 CAF *M* = 171.3, SD = 48.39, F3 CON *M* = 164.5, SD = 55.25; *t*_(21)_ = 0.529 *p* = 0.5980, *d* = 0.153). Notably, F2 CAF rats showed increased immobility time (F2 CAF group *M* = 209.2, SD = 43.81; F2 CON group *M* = 152.5, SD = 56.47; Fig. 3*E*; *t*_(14)_ = 2.254 *p = 0.0407, *d* = 1.020) and less swimming time (*M* = 59, SD = 30.67) when compared with the F2 CON (*M* = 117.5, SD = 68.67) during the FST (Fig. 3*F*; *t*_(15)_ = 2.316 *p = 0.0351, *d* = 1.536). Finally, contrary to the behavioral depression-like outcomes found in the F1 and F2 rats during the swimming test, the F3 rats born from individuals exposed to CAF diet showed less immobility time (Fig. 3*H*; F3 CAF group *M* = 134.5, SD = 60.58; F3 CON group *M* = 178.9, SD = 58.91; *t*_(67)_ = 3.021 **p = 0.0036, *d* = −0.758) and more swimming time compared with the F3 rats exposed to CON diet (Fig. 3*I*; F3 CAF group *M* = 150, SD = 55.92; F3 CON group *M* = 110.4, SD = 58.3; *t*_(67)_ = 2.84 **p = 0.006, *d* = 0.679). Together, depression-like behavior primed by prenatal and lactation exposure to CAF diet is preserved until the F2 offspring generation as an intergenerational inheritance.

**Figure 4.**
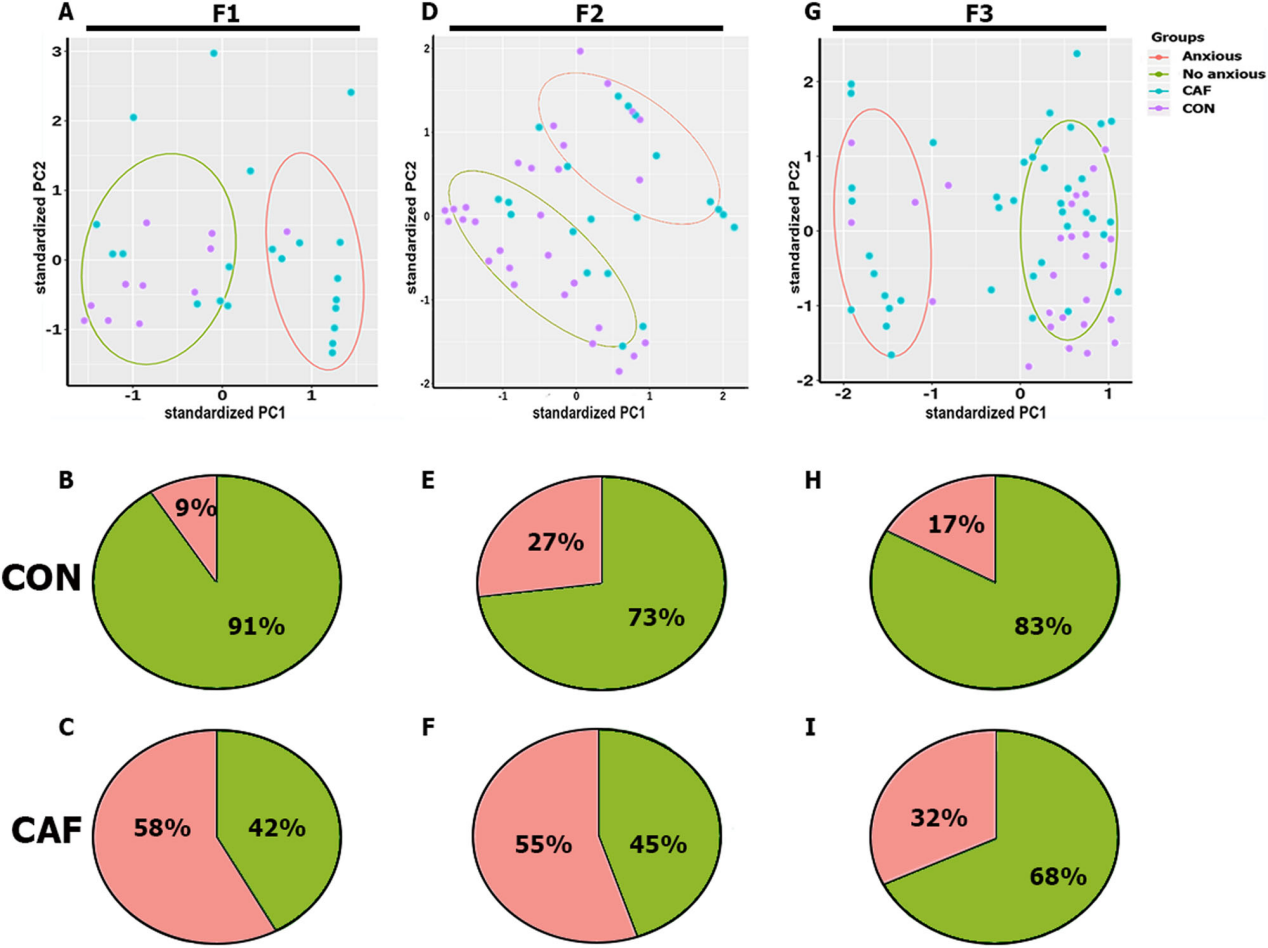
Behavioral phenotype characterization using PCA. PCA biplot of clustering nonanxious (green line) and anxious subjects (red line) from CAF (blue dots) and CON (purple dots) group in (***A***) F1, (***D***) F2, and (***G***) F3 generations. Phenotype pie charts describing the percentage of nonanxious and anxious subjects of each generation and diet group F1 (***B***,***C***), F2 (***E***,***F***), and F3 (***H***,***I***). The representative image depicting the inheritance of the phenotype based on PCA results contribution can be found in Extended Data Figure 4-1, while Extended Data Figure 4-2 contains the PCA contributions of variables.

Next, we applied a PCA analysis to classify behavioral outcomes as anxiety (A) and nonanxiety (NA) in the F1, F2, and F3 rats dictated by diet exposure (CON vs CAF diet) during the prenatal stage (Extended Data Fig. 4-1, see color labels for details). It is worth noting that initial PCA results showed that the depression test parameters did not contribute to the clustering (Extended Data Fig. 4-2), and therefore, they were excluded from further analysis. Only anxiety-related parameters were utilized for subject classification. The PC1 versus PC2 analysis showed that 90.91% of the F1 rats born from dams exposed to CON diet and 42.86% of F1 rats born from dams exposed to CAF diet were classified as showing nonanxiety-like behavior. Conversely, prenatal exposure to CAF diet accounts for up to 57.14% of F1 offspring with anxiety-like behavior, whereas just 9.09% of F1 offspring were found in subjects exposed to the control diet (Fig. 4*A–C*). Accordingly, we found that F1 rats prenatally and during lactation exposed to CAF diet were more likely than F1 CON rats to have anxiety-like behavior phenotype [*X*^2^(1, *N* = 31) = 6.910, **p = 0.0086].

10.1523/ENEURO.0194-23.2023.f4-1 Figure 4-1 Color-coded representation of each behavioral phenotype accordingly to each generation and diet group. Download Figure 4-1, TIF file.

10.1523/ENEURO.0194-23.2023.f4-2 Figure 4-2 PCA contribution of variables to variance in A) F1, B) F2 and C) F3. Download Figure 4-2, TIF file Figure 4-2, DOCX file.

The F2 and F3 rats born from the F1 generation prenatally and during lactation exposed to CAF diet showed more individuals with anxiety-like behavior (Fig. 4*D–I*). Accordingly, the PCA analysis identified that up to 55% of F2 individuals born from the F1 rats exposed to CAF diet were with anxiety-like behavior, when compared with 27% of F2 individuals born from the F1 rats exposed to the control diet (Fig. 4*D–F*). In contrast, 72.41% of F2 rats born from the control F1 individuals and 45% of F2 individuals born from the F1 rats exposed to CAF diet, displayed nonanxiety-like behavior [Fig. 4*D–F*; *X*^2^(1, *N* = 51) = 5.148, *p = 0.0233].

Finally, the F3 rats born from the F1 subjects exposed to CAF diet significantly preserved the anxiety-like behavior, by showing 32.50% of individuals whereas 17.24% of F3 offspring born from the F1 subjects were exposed to the control diet (Fig. 4*G–I*). Also, up to 82.76% and 67.50% of F3 rats born from the F1 rats exposed to control or CAF diet, respectively, were classified with nonanxiety-like behavior [*X*^2^(1, *N* = 70) = 2.030, *p* = 0.1542] (Fig. 4*G–I*). These results confirmed that prenatal and lactation exposure to CAF diet primed the transgenerational inheritance of anxiety-like behavior in the offspring.

### Transgenerational inheritance of anxiety-like behavior in the offspring relates to brain macrostructural alterations

We performed DBM analysis to identify potential macrostructural local volume changes in brain regions of F1, F2, and F3 rats with anxiety-like behavior. We found significant differences between groups within generations in specific brain regions (Tables 1–4). To identify macrostructural brain changes regarding the anxiety-like behavior phenotype, we first compared brain volume changes between control individuals showing anxiety-like behavior (CON-A) versus nonanxious control individuals (CON-NA). We found that the F1 CON-A offspring showed higher volume in the right piriform cortex compared with the F1 CON-NA offspring (Table 1). We did not find significant differences in brain volume when comparing the F2 CON-A offspring versus F2 CON-NA rats. However, we found higher volume in the caudate putamen, frontal association cortex, and retrosplenial cortex and lower volume in the amygdala, thalamus, primary somatosensory cortex, and dentate gyrus of the F3 CON-A rats when compared with the F3 CON-NA offspring (Table 1). Next, we determined the effect of prenatal and lactation exposure to CAF diet on the transgenerational inheritance of macrostructural brain alterations by comparing CON-NA individuals versus individuals prenatally and during lactation exposed to CAF diet and exhibiting anxiety-like behavior (CAF-A) for the F1, F2, and F3 generations. We identified that the F1 CAF-A rats showed higher volume in cerebellar lobule 3, right thalamus, and right secondary motor cortex and lower volume in right hippocampal layer CA3, left frontal association cortex, cerebellar lobule 6, and left secondary somatosensory cortex when compared with the F1 CON-NA offspring (Table 2). We did not find significant differences in brain volume that survived the FDR correction when comparing the F2 CAF-A versus F2 CON-NA offspring. Finally, the F3 CAF-A rats showed higher volume in the third ventricle, copula, rhinal cortex, and amygdala and lower volume in the internal capsule, retrosplenial cortex, and lateral ventricle (LV) when compared with the F3 CON-NA offspring (Table 2).

**Table 1.** Volumetric changes in brain regions comparing the CON-NA versus CON-A from F1, F2, and F3 offspring

Generation	Label	*t* value	*x*	*y*	*z*
F1	Right piriform cortex	11.93	6.3395	−2.7505	3.3544
F3	Caudate putamen	5.595	−4.7604	−8.1505	3.1744
	Caudate putamen	4.782	4.7195	−5.4505	5.8744
	Frontal association cortex	4.388	1.9595	−12.0505	5.0944
	Retrosplenial cortex	4.363	−0.6204	−3.8305	8.6944
	Amygdaloid area	−5.713	3.0995	−6.7105	2.0944
	Thalamus	−5.657	−2.4804	−5.9305	5.6344
	Primary somatosensory cortex	−5.557	5.1995	−7.7905	7.1344
	Crus 1 ansiform lobule	−4.797	4.5395	2.8294	7.8544
	CC and associated white matter	−4.456	−3.1404	−2.0905	9.1744
	Cerebellar white matter/arbor vitae of the cerebellum	−4.431	−1.8804	4.7494	7.4944
	Dentate gyrus	−4.263	3.9995	−2.9305	6.7744

Volumetric changes in brain regions according to the XYZ coordinates based on the Fisher 344 atlas.

**Table 2.** Volumetric changes in brain regions comparing the CON-NA versus CAF-A from F1, F2, and F3 offspring

Generation	Label	*t* value	*x*	*y*	*z*
F1	Cerebellar lobule 3	12.31	−1.2804	−9.9505	8.8144
	Right thalamus	10.65	3.5795	−5.1505	5.3944
	Right secondary motor cortex	8.471	0.3395	−2.8105	10.6745
	Cerebellar white matter/arbor vitae of the cerebellum	6.733	4.1195	−12.3505	5.6344
	Right hippocampal layer CA3	−13.36	3.8195	−4.9705	7.3744
	Left frontal association cortex	−10.06	−1.6404	2.8894	6.5944
	Cerebellar lobule 6	−9.749	0.2195	−12.4705	9.1144
	Left secondary somatosensory cortex	−8.394	−8.1204	−1.1905	6.1144
F3	Third ventricle	6.412	−1.1604	−8.0905	5.8744
	Copula	5.872	−4.4604	4.0894	5.0944
	Rhinal cortex	5.771	5.3195	0.0694	4.9144
	Amygdaloid area	5.13	−4.7004	−6.8905	1.7944
	Hippocampal layer CA3	5.124	3.2195	−3.9505	2.6944
	Cerebellar white matter/arbor vitae of the cerebellum	4.875	1.2395	4.8694	8.2744
	Internal capsule	−5.267	2.5595	−5.3905	3.2344
	Retrosplenial cortex	−5.243	−2.6004	−2.1505	9.9544
	LV	−5.098	−4.4604	−5.1505	3.2344
	Cerebellar lobule 6	−5.086	0.3395	3.7294	9.1744
	CC and associated white matter	−5.001	−4.2804	−10.9105	6.0544

Volumetric changes in brain regions according to the XYZ coordinates based on the Fisher 344 atlas.

Next, to identify changes in brain regions dictated by prenatal diet on the transgenerational inheritance of anxiety-like behavior, we compared the effect of diet on each behavioral outcome in the F1, F2, and F3 generations according to the PCA analysis. Initially, we compared the effect of prenatal diet exposure (CON vs CAF) in offspring exhibiting a nonanxious phenotype (CON-NA vs CAF-NA). We found that in contrast to the nonanxious F1 rats exposed to control diet (F1 CON-NA), the nonanxious F1 rats exposed to CAF diet (F1 CAF-NA) displayed higher volume in cerebellar lobule 3, right olfactory bulb, and right hypothalamus and lower volume in the left infralimbic cortex, left substantia nigra, right Crus 1 ansiform lobule, left secondary somatosensory cortex, and right thalamus (Table 3). Again, we did not find significant differences that survived the FDR correction in specific brain regions between F2 CAF-NA offspring and F2 CON-NA offspring. Moreover, the F3 CAF-NA offspring exhibited higher volume in the third ventricle, hypothalamus, and paramedial lobule and lower volume in the CC and associated white matter, subincular region, and internal capsule when compared with the F3 CON-NA offspring (Table 3). Finally, we compared the effect of prenatal diet exposure (CON vs CAF) in rats exhibiting an anxious phenotype. We found that in contrast to the anxious F1 rats exposed to control diet (F1 CON-A), the anxious F1 rats exposed to CAF diet (F1 CAF-A) displayed higher volume in cerebellar lobule 3, right primary somatosensory cortex, and right amygdaloid area and lower volume in right piriform cortex, right hippocampal layer CA3, left frontal association cortex, amygdala, and periaqueductal gray (Table 4). Accordingly, higher volume in the right hypothalamus, paramedian lobule, and secondary somatosensory cortex and lower brain volume in the LV and right dentate gyrus was found in the F2 CAF-A rats compared with the F2 CON-A rats (Table 4). Also, the F3 CAF-A rats showed higher volume in the paramedian lobule, primary somatosensory cortex, and amygdaloid area, whereas they showed lower volume in the amygdaloid area, internal capsule, and cerebellar lobules 4 and 5 compared with the F3 CON-A rats (Table 4).

**Table 3.** Effect of diet on volumetric changes in brain regions comparing CAF-NA versus CON-NA from F1, F2, and F3 offspring

Generation	Label	*t* value	*x*	*y*	*z*
F1	Cerebellar lobule 3	17.38	−1.2204	−9.9505	8.8144
	Right olfactory bulb	13.91	0.7595	6.2494	9.8344
	Right hypothalamus	12.35	0.4595	−2.6305	3.1144
	Left olfactory bulb	8.073	−1.8204	3.9094	3.5344
	left infralimbic cortex	−17.72	−1.6404	2.7694	6.8344
	Left substantia nigra	−13.8	−2.7204	−4.8505	3.2344
	Right crus 1 ansiform lobule	−12.42	5.4395	−11.8105	6.3544
	Right thalamus	−10.29	0.6995	−1.6705	6.5944
	Left secondary somatosensory cortex	−9.287	−8.1204	−1.2505	6.1144
	Cerebellar lobule 6	−9.135	0.2195	−12.4705	9.1144
F3	Third ventricle	5.543	−0.6204	−4.6705	6.2344
	Hypothalamus	5.035	0.0804	−8.3905	2.5744
	Paramedian lobe	4.952	3.5195	4.1494	6.3544
	Subincular region	4.921	5.5595	−2.4505	3.7744
	Flocculus	4.678	−7.4604	2.3494	4.2544
	Cerebellar lobule 8	4.464	−1.6404	5.2294	7.0144
	Cingulate cortex area 1	3.952	0.3995	−12.7105	8.2744
	Hippocampal layer CA1	3.872	−5.5404	−3.1705	4.7344
	CC and associated white matter	−5.446	1.0595	−11.3905	5.2744
	CC and associated white matter	−5.324	1.8995	−5.0905	9.2944
	Subicular region	−5.236	−4.9404	−0.2905	7.1944
	Internal capsule	−5.223	2.5595	−5.3305	3.2944
	Stria terminalis	−5.025	−4.8204	−6.2905	6.4744
	Secondary visual cortex	−4.586	5.4395	−1.0705	8.8744
	Piriform cortex	−4.359	−6.4404	−9.1105	2.6344
	Rhinal cortex	−4.169	6.1595	−1.1305	3.3544
	Olfactory bulb	−4.152	−3.5004	−13.7305	4.6744

Volumetric changes in brain regions according to the XYZ coordinates based on the Fisher 344 atlas.

**Table 4.** Effect of diet on volumetric changes in brain regions comparing CAF-A versus CON-A from F1, F2, and F3 offspring

Generation	Label	*t* value	*x*	*y*	*z*
F1	Cerebellar lobule 3	14.64	−1.2204	−9.9505	8.814496
	Right primary somatosensory cortex	10.01	2.8595	2.8894	6.594496
	Right crus 1 ansiform lobule	9.569	5.4395	−11.8105	6.354496
	Left paraflocculus	8.872	−6.4404	−11.2705	4.074496
	Right amygdaloid area	7.913	3.4595	−2.4505	1.734496
	Right olfactory bulb	7.386	0.7595	6.2494	9.834496
	Right primary somatosensory cortex	6.901	5.6795	−2.9905	8.754496
	Right piriform cortex	−10.2	6.3995	−3.0505	3.174496
	Right hippocampal layer CA3	−9.099	3.6395	−5.0305	7.254496
	Left frontal association cortex	−8.177	−1.7004	3.0694	6.894496
	Amygdala	−8.096	−5.4804	−3.1105	4.074496
	Periaqueductal gray	−6.963	−1.6404	−7.6705	5.574496
F2	Right hypothalamus	6.375	0.2795	−3.6505	4.854496
	Paramedian lobule	5.264	2.4995	4.5094	6.654496
	Secondary somatosensory cortex	5.11	5.1395	−7.7905	4.434496
	Left motor cortex	5.015	−2.2404	−9.7105	8.094496
	CC and associated white matter	4.886	1.6595	−6.1705	8.994496
	CC and associated white matter	4.543	−3.4404	−3.1705	9.234496
	LV	−5.78	−5.1804	−5.0305	4.434496
	Right dentate gyrus	−5.415	3.4595	−6.1705	7.074496
F3	Paramedian lobe	6.158	1.1195	3.5494	6.894496
	Primary somatosensory cortex	6.048	5.1995	−7.7905	7.134496
	Amygdaloid area	5.913	−3.9804	−6.4105	1.734496
	Amygdaloid area	5.332	3.0995	−6.7105	2.094496
	Rhinal cortex	5.251	5.1395	−0.0505	5.814496
	Thalamus	4.729	−1.4004	−5.1505	6.174496
	Frontal association cortex	4.72	2.1995	−11.7505	3.414496
	Paraflocculus	4.594	−4.8204	3.3094	5.514496
	Primary somatosensory cortex	4.515	−3.5604	−9.9505	8.514496
	Amygdaloid area	−5.757	−3.9804	−6.5905	2.514496
	Internal capsule	−5.26	3.2795	−6.5305	4.614496
	Cerebellar lobules 4 and 5	−5.248	−0.1404	2.8894	9.894496
	Lateral septum	−5.053	−1.4604	−9.3505	6.594496
	Retrosplenial cortex	−4.671	−0.6204	−3.7705	8.574496
	Frontal association cortex	−4.536	2.0795	−13.0705	5.514496
	Frontal association cortex	−4.395	−3.7404	−14.6905	6.774496

Volumetric changes in brain regions according to the XYZ coordinates based on the Fisher 344 atlas.

In our study, we employed a two-way ANOVA to investigate the interplay between prenatal diet and phenotype in relation to local brain volume for the F1, F2, and F3 generations. Our analysis revealed a significant interaction between these two factors, as presented in Tables 5–7. Our primary focus was on exploring the main effects of prenatal diet exposure and anxiety phenotype on brain structure.

**Table 5.** Results of two-way ANOVA for prenatal diet and behavioral phenotype group comparison in brain region of F1 corresponding to Figure 5

Region	Model		Degree of freedom	Sum of squares	Mean square	*F* value	Adjusted *p*-value
CA3 Contrast CON-NA Vs CAF-A	CA3 ∼ PD * phenotype	PD	1	0.0329	0.0329	105.960	0.0001***
		Phenotype	1	0.0114	0.0114	36.791	0.0001***
		PD * phenotype	1	0.0002	0.0002	0.945	0.3539
Lb3 Contrast CON-A Vs CAF-A	Lb3 ∼ PD * phenotype	PD	1	0.1964	0.1964	429.879	0.0001***
		Phenotype	1	0.0339	0.0339	74.367	0.0001***
		PD * phenotype	1	0.0038	0.0038	8.459	0.0156*
Lb3 Contrast CON-NA Vs CAF-A	Lb3 ∼ PD * phenotype	PD	1	0.1964	0.1964	429.879	0.0001***
		Phenotype	1	0.0339	0.0339	74.367	0.0001***
		PD * phenotype	1	0.0038	0.0038	8.459	0.0156*
Lb3 Contrast CON-NA Vs CAF-NA	Lb3 ∼ PD * phenotype	PD	1	0.1964	0.1964	429.879	0.0001***
		Phenotype	1	0.0339	0.0339	74.367	0.0001***
		PD * phenotype	1	0.0038	0.0038	8.459	0.0156*
TH Contrast CON-NA Vs CAF-A	TH ∼ PD * phenotype	PD	1	0.0094	0.0094	10.069	0.0099**
		Phenotype	1	0.0258	0.0258	27.432	0.0003***
		PD * phenotype	1	0.0080	0.0080	8.565	0.01513*
IL Contrast CON-NA Vs CAF-NA	IL ∼ PD * phenotype	PD	1	0.1412	0.1412	257.68	0.0001***
		Phenotype	1	0.0450	0.0450	82.16	0.0001***
		PD * phenotype	1	0.0056	0.0056	10.26	0.0094*
HT Contrast CON-NA Vs CAF-NA	HT ∼ PD * phenotype	PD	1	0.0242	0.0242	58.855	0.0001***
		Phenotype	1	0.0005	0.0005	1.358	0.2710
		PD * phenotype	1	0.0015	0.0015	3.684	0.0839
PIR Contrast CON-NA Vs CON-A	PIR ∼ PD * phenotype	PD	1	0.0001	0.0001	0.072	0.7935
		Phenotype	1	0.0378	0.0378	19.724	0.0012**
		PD * phenotype	1	0.1422	0.1422	74.166	0.0001***

Behavioral traits in the offspring of mice prenatally exposed to HED. PD = distance traveled during open field.

* <0.05.

** <0.01.

*** <0.001.

**Table 6.** Results of two-way ANOVA for prenatal diet and behavioral phenotype group comparison in brain region of F2 corresponding to Extended Data Figure 4-2

Region	Model		Degree of freedom	Sum of squares	Mean square	*F* value	Adjusted *p*-value
PML Contrast CON-A Vs CAF-A	PML ∼ PD * phenotype	PD	1	0.0497	0.0497	19.820	0.0001***
		Phenotype	1	0.0114	0.0114	4.541	0.0396*
		PD * phenotype	1	0.0345	0.0345	13.787	0.0006***
FrA Contrast CON-A Vs CAF-A	FrA ∼ PD * phenotype	PD	1	0.1695	0.1695	16.343	0.0002***
		Phenotype	1	0.0048	0.0048	0.463	0.5005
		PD * phenotype	1	0.1168	0.1167	11.259	0.0018**
HT Contrast CON-A Vs CAF-A	HT ∼ PD * phenotype	PD	1	0.0280	0.0280	26.523	0.0001***
		Phenotype	1	0.0035	0.0035	3.314	0.0765
		PD * phenotype	1	0.0166	0.0166	15.730	0.0003***

Behavioral traits in the offspring of mice prenatally exposed to HED. PD = distance traveled during open field.

* <0.05.

** <0.01.

*** <0.001.

**Table 7.** Results of two-way ANOVA for prenatal diet and behavioral phenotype group comparison in brain region of F3 corresponding to Figure 6

Region	Model		Degree of freedom	Sum of squares	Mean square	*F* value	Adjusted *p*-value
AMY Contrast CON-A Vs CAF-A	AMY ∼ PD * phenotype	PD	1	0.1138	0.1138	15.067	0.0003***
		Phenotype	1	0.0000	0.0000	0.000	0.9943
		PD * phenotype	1	0.0682	0.0682	9.032	0.0041**
S1 Contrast CON-A Vs CAF-A	S1 ∼ PD * phenotype	PD	1	0.0089	0.0089	5.01	0.0297*
		Phenotype	1	0.0001	0.0001	0.07	0.7921
		PD * phenotype	1	0.0181	0.0181	10.15	0.0025**
3V Contrast CON-NA Vs CAF-A	3V ∼ PD * phenotype	PD	1	0.1096	0.1096	26.945	0.0001***
		Phenotype	1	0.0164	0.0164	4.050	0.0497*
		PD * phenotype	1	0.0127	0.0127	3.131	0.0830
RCx Contrast CON-NA Vs CAF-A	RCx ∼ PD * phenotype	PD	1	0.0928	0.0928	15.79	0.0002***
		Phenotype	1	0.0678	0.0678	11.54	0.0013**
		PD * phenotype	1	0.0891	0.0891	15.17	0.0002***
3V Contrast CON-NA Vs CAF-NA	3V ∼ PD * phenotype	PD	1	0.1236	0.1236	13.933	0.0004***
		Phenotype	1	0.0052	0.0052	0.591	0.4458
		PD * phenotype	1	0.1590	0.1590	17.924	0.0001***
CC Contrast CON-NA Vs CAF-NA	CC ∼ PD * phenotype	PD	1	0.2089	0.2089	16.790	0.0001***
		Phenotype	1	0.0132	0.0132	1.063	0.3076
		PD * phenotype	1	0.1207	0.1207	9.700	0.0030**
HT Contrast CON-NA Vs CAF-NA	HT ∼ PD * phenotype	PD	1	0.0536	0.0536	7.470	0.0087**
		Phenotype	1	0.0006	0.0006	0.088	0.7678
		PD * phenotype	1	0.0805	0.0804	11.213	0.0015**
CPu Contrast CON-NA Vs CON-A	CPu ∼ PD * phenotype	PD	1	0.0285	0.0284	3.861	0.0551
		Phenotype	1	0.0804	0.0803	10.902	0.0017**
		PD * phenotype	1	0.1311	0.1311	17.790	0.0001***
AMY Contrast CON-NA Vs CON-A	AMY ∼ PD * phenotype	PD	1	0.0104	0.0104	2.258	0.1394
		Phenotype	1	0.0232	0.0232	5.033	0.0294*
		PD * phenotype	1	0.1072	0.1072	23.261	0.0001***
FrA Contrast CON-NA Vs CON-A	FrA ∼ PD * phenotype	PD	1	0.0214	0.0214	5.486	0.0232*
		Phenotype	1	0.0361	0.0361	9.246	0.0037**
		PD * phenotype	1	0.0364	0.0364	9.344	0.0036**
PML Contrast CON-NA Vs CON-A	FrA ∼ PD * phenotype	PD	1	0.0673	0.0673	17.423	0.0001***
		Phenotype	1	0.0036	0.0036	0.946	0.3354
		PD * phenotype	1	0.0685	0.0685	17.735	0.0001***

Behavioral traits in the offspring of mice prenatally exposed to HED. PD = distance traveled during open field.

* <0.05.

** <0.01.

*** <0.001.

**Figure 5.**
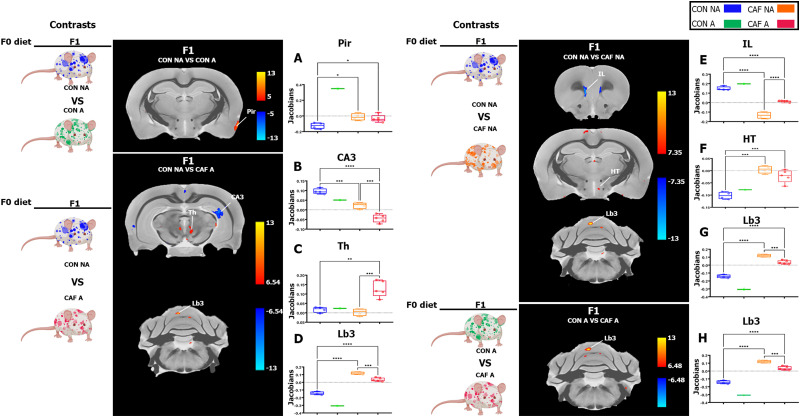
Maternal HED effect on brain volume in the F1 offspring analyzed by DBM. Representative coronal MRI section showing affected areas that were found to increase (red) or decrease (blue) among each contrast comparison color-coded as the phenotype group alongside the box plot showing volume differences (*y*-axis = Jacobians) in each group (*x*-axis) in specific region with higher significant changes in DBM analysis. F1 Jacobian values were compared in (***A***) PIR, (***B***) CA3, (***C***) Th, (***D***) Lb3, (***E***) IL, (***F***) HT, (***G***) Lb3, and (***H***) Lb3. The results are expressed as mean ± SEM, followed by ANOVA post hoc Tukey. *p < 0.05. CON-NA, nonanxious control diet group; CON-A, anxious control diet group; CAF-NA, nonanxious CAF diet group; CAF-A, anxious CAF diet group. To compare body weights across different groups and examine the results of volume changes within the F2 generation, please refer to Extended Data Figure 5-1 and Extended Data Figure 5-2, respectively.

Additionally, we conducted pairwise group comparisons to pinpoint specific brain regions influenced by prenatal diet exposure and anxiety phenotype. This method afforded us the ability to accurately ascertain the impact of each variable on regional brain volumes, a crucial undertaking given the extensive number of brain regions under scrutiny. We found a significant higher volume in the Jacobian value of piriform cortex in F1 CAF-NA compared with the F1 CON-NA group (Table 5; Fig. 5*A*). Also, we identified a lower volume in hippocampal layer CA3 significant peak in F1 CAF-NA and F1 CAF-A compared with F1 CON-NA group (Table 5; Fig. 5*B*), higher volume in the thalamus (Th) significant peak in F1 CAF-A compared with F1 CON-NA group (Table 5; Fig. 5*C*), and higher volume in cerebellar lobule 3 (Lb3) significant peak in F1 CAF-NA and F1 CAF-A compared with the F1 CON-NA group (Table 5; Fig. 5*D*).

10.1523/ENEURO.0194-23.2023.f5-1 Figure 5-1 Boxplot comparing body weights across groups categorized by F0 diet (CON, CAF), behavioral phenotype, and offspring A) F1, B) F2, and C) F3. Significant differences between groups are indicated by asterisks, with * p < 0.05, ** p < 0.01, and *** p < 0.001. Post hoc Tukey's analysis was utilized to determine specific group differences. Download Figure 5-1, TIF file.

10.1523/ENEURO.0194-23.2023.f5-2 Figure 5-2 Representative coronal MRI section showing affected areas that were found to increase (red) or decrease (blue) among each contrast (CON A vs CAF A) comparation color-coded as the phenotype group alongside the box plot showing volume differences (y axis = Jacobians) in each group (x axis) in specific region with higher significant changes in DBM analysis, whereas F2 Jacobian values was compared in A) LV, B) HT C) PML. Results are expressed as mean + SEM, following by ANOVA post hoc Tukey. *p<0.05. Download Figure 5-2, TIF file.

Next, we found lower volume in the infralimbic cortex (IL) peak in F1 CAF-NA and F1 CAF-A compared with the F1 CON-NA group (Table 5, Fig. 5*E*), higher volume in the hypothalamus (HT) peak in F1 CAF-NA and F1 CAF-A compared with the F1 CON-NA group (Table 5, Fig. 5*F*), and higher volume in the cerebellar lobule 3 (Lb3) significant peak in F1 CAF-NA and F1 CAF-A compared with the F1 CON-NA group (Table 5; Fig. 5*G*). We also found a higher volume in the cerebellar lobule 3 (Lb3) significant peak in F1 CAF-NA and F1 CAF-A compared with the F1 CON-NA group (Table 5, Fig. 5*H*).

Furthermore, we compared the extracted Jacobian from brain significant peaks of the F2 generation. We identified a major significant effect when comparing CON-A versus CAF-A (Table 4). We found that F2 CAF-A offspring showed lower volume in the LV peak (Table 6, Extended Data Fig. 5-2A), higher volume in hypothalamus (HT) peak compared with the F2 CON-A (Table 6, Extended Data Fig. 5-2B), and higher volume in paramedian lobule (PML) peak (Table 6, Extended Data Fig. 5-2C).

**Figure 6.**
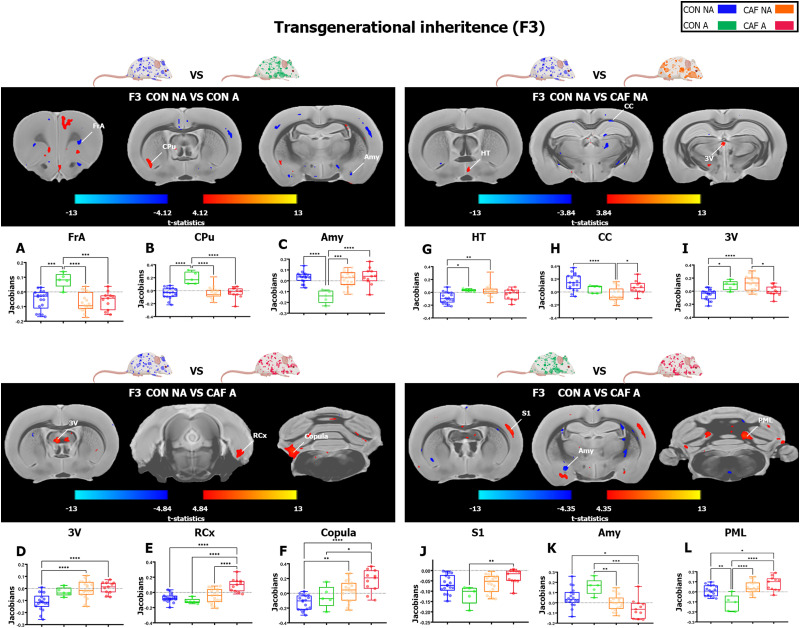
Transgenerational inheritance effect of maternal HED on brain volume analyzed by DBM in the F3 offspring. Representative coronal MRI section showing affected areas that were found to increase (red) or decrease (blue) among each contrast comparison color-coded as the phenotype group alongside the box plot showing volume differences (*y*-axis = Jacobians) in each group (*x*-axis) in specific region with higher significant changes in DBM analysis, whereas F3 Jacobian values was compared in (***A***) FrA, (***B***) CPu, (***C***) Amy, (***D***) 3V, (***E***) RCx, (***F***) copula, (***G***) HT, (***H***) CC, (***I***) 3V, (***J***) S1, (***K***) Amy, and (***L***) PML. The results are expressed as mean ± SEM, followed by ANOVA post hoc Tukey. *p < 0.05. CON-NA, nonanxious control diet group; CON-A, anxious control diet group; CAF-NA, nonanxious CAF diet group; CAF-A, anxious CAF diet group.

Finally, we compared the extracted Jacobian from brain significant peaks of the F3 generation. First, we found lower volume in the frontal association cortex (FrA) peak in CON-NA, CAF-NA, and CAF-A compared with the CON-A group (Table 7, Fig. 6*A*), lower volume in caudate–putamen (Cpu) peak in CON-NA, CAF-NA, and CAF-A compared with the CON-A group (Table 7, Fig. 6*B*) and higher volume in the amygdaloid area (Amy) peak in CON-NA, CAF-NA, and CAF-A compared with the CON-A group (Table 7, Fig. 6*C*). Next, we found that CAF-NA and CAF-A showed higher volume in the third ventricle (3V) peak compared with the CON-NA group (Table 7, Fig. 6*D*), CAF-A showed higher volume in the rhinal cortex peak (RCx) compared with CON-NA (Table 7, Fig. 6*E*), and higher volume in the copula peak (Table 7, Fig. 6*F*).

Finally, we found that CON-A and CAF-NA showed higher volume in the hypothalamus (HT) peak compared with the CON-NA group (Table 7, Fig. 6*G*). On the other hand, CAF-NA showed lower volume in the CC peak compared with the CON-NA group and CAF-A showed higher volume compared with the CAF-NA group (Table 7, Fig. 6*H*). Conversely, CON-A and CAF-NA showed higher volume in the third ventricle (3V) peak compared with the CON-NA offspring (Table 7, Fig. 6*I*) and CAF-A showed lower volume compared with the CAF-NA individuals (Table 7, Fig. 6*I*). Moreover, we found that CAF-A showed higher volume in the somatosensory cortex (S1) peak compared with the CON-A group (Table 7, Fig. 6*J*). Also, we found significant differences in the volume of the amygdaloid (Amy) area peak among the groups (Table 7, Fig. 6*K*), where CAF-NA and CAF-A showed lower volume compared with CON-A group (Table 7, Fig. 6*K*) and CAF-A showed lower volume compared with the CON-NA group (Fig. 6*K*). Lastly, we found significant differences in the volume of the paramedian lobule peak (PML) among the groups (Table 7, Fig. 6*L*), where CAF-NA and CAF-A showed higher volume compared with the CON-A group (Table 7, Fig. 6*L*), only CAF-A showed higher volume compared with the CON-NA group (Table 7, Fig. 6*L*), and CON-A showed lower volume compared with the CON-NA group (Fig. 6*L*).

Together, these results confirm the transgenerational inheritance of macrostructural alterations in the rats prenatally and during lactation exposed to an HED, which relates to anxiety-like behavior.

### Transgenerational inheritance of anxiety-like behavior in the offspring correlates with brain microstructural alterations

**Figure 7.**
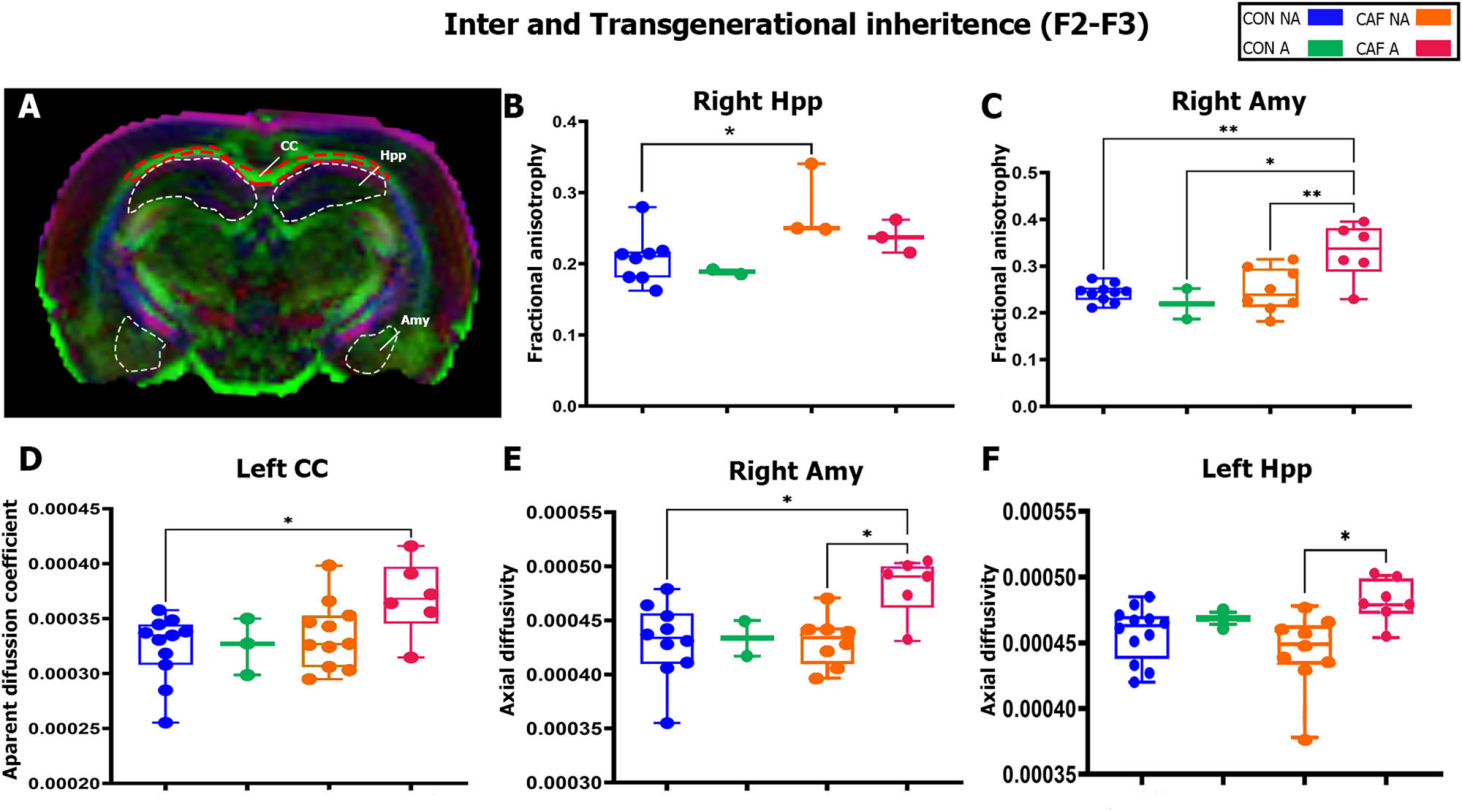
Inter- and transgenerational changes in DWI. Representative vector map showing ROIs from where diffusion metric FA, AD, and ADC values were extracted. Box plot showing differences in (***B***) F2 offspring FA in right Hpp, (***C***) FA in right Amy, (***D***) ADC in left CC, (***E***) AD in right Amy, and (***F***) AD in left Hpp. The results are expressed as mean ± SEM, followed by ANOVA post hoc Tukey. *p < 0.05. The comprehensive results of the comparisons among all regions and diffusion variables are detailed in Extended Data Tables 7-1 to 7-9. CON-NA, nonanxious control diet group; CON-A, anxious control diet group; CAF-NA, nonanxious CAF diet group; CAF-A, anxious CAF diet group.

We were interested in characterizing the global microstructural changes in the F1, F2, and F3 rats exhibiting an anxiety-like behavior. We used the DTI analysis integrating the FA, AD, ADC, and RD values for each brain region in the F1, F2, and F3 rats. Initially, F1 rats prenatally and during lactation exposed to CAF diet did not change the FA maps when compared with the F1 offspring exposed to the control diet (Extended Data Table 7-1). In contrast, F2 CAF-NA offspring showed a significant increase in the right hippocampus (Extended Data Table 7-2, Fig. 7*B*; *F*_(3,12)_ = 3.79, *p* = 0.04) compared with the F2 CON-NA offspring (Fig. 7*B*; F2 CON-NA vs F2 CAF-NA *p = 0.0489). Notably, major changes in the FA maps were found in the amygdala of F3 offspring according to prenatal diet exposure (Extended Data Table 7-3; Fig. 7*C F*_(3,22)_ = 6.983, *p* = 0.001). Precisely, the F3 CAF-A offspring showed an increase in the FA value of the right amygdala when compared with the F3 CON-NA offspring (Fig. 7*C*; **p = 0.0028), with the F3 CON-A offspring (Fig. 7*C*; *p = 0.018), or with the F3 CAF-NA offspring (Fig. 7*C*; **p = 0.007). The F3 CAF-A offspring also displayed a significant increase in the ADC value in the left CC (Extended Data Table 7-6; Fig. 7*D*; *F*_(3,27)_ = 3.011, *p* = 0.047) compared with the F3 CON-NA offspring (Fig. 7*D*; *p = 0.034). Accordingly, the F3 CAF-A offspring showed a significant increase in the AD value of the right amygdala (Extended Data Table 7-9; Fig. 7*E*; *F*_(3,22)_ = 4.452, *p* = 0.013) compared with the F3 CON-NA offspring (Fig. 7*E*; *0.015) and to the F3 CAF-NA offspring (Fig. 7*E*; *p = 0.022). Finally, a significant increase in AD value was found in the left hippocampus of F3 CAF-A offspring (Extended Data Table 7-9; Fig. 7*F*; *F*_(3,26)_ = 3.806, *p* = 0.021) when compared with the F3 CAF-NA offspring (Fig. 7*F*; *p = 0.013). Together, these results demonstrate that maternal HED has a transgenerational effect on microstructural alteration.

10.1523/ENEURO.0194-23.2023.t7-1 Table 7-1 p- values from FA comparation between CON-NA vs CON-A, CAF-NA and CAF-A; CON-A vs CAF-NA, CAF-A; and CAF-NA vs CAF-A in the F1 offspring. Download Table 7-1, DOCX file.

10.1523/ENEURO.0194-23.2023.t7-2 Table 7-2 p- values from FA comparation between CON-NA vs CON-A, CAF-NA and CAF-A; CON-A vs CAF-NA, CAF-A; and CAF-NA vs CAF-A in the F2 offspring. Download Table 7-2, DOCX file.

10.1523/ENEURO.0194-23.2023.t7-3 Table 7-3 p- values from FA comparation between CON-NA vs CON-A, CAF-NA and CAF-A; CON-A vs CAF-NA, CAF-A; and CAF-NA vs CAF-A in the F3 offspring. Download Table 7-3, DOCX file.

10.1523/ENEURO.0194-23.2023.t7-4 Table 7-4 p- values from ADC comparation between CON-NA vs CON-A, CAF-NA and CAF-A; CON-A vs CAF-NA, CAF-A; and CAF-NA vs CAF-A in the F1 offspring. Download Table 7-4, DOCX file.

10.1523/ENEURO.0194-23.2023.t7-5 Table 7-5 p- values from ADC comparation between CON-NA vs CON-A, CAF-NA and CAF-A; CON-A vs CAF-NA, CAF-A; and CAF-NA vs CAF-A in the F2 offspring. Download Table 7-5, DOCX file.

10.1523/ENEURO.0194-23.2023.t7-6 Table 7-6 p- values from ADC comparation between CON-NA vs CON-A, CAF-NA and CAF-A; CON-A vs CAF-NA, CAF-A; and CAF-NA vs CAF-A in the F3 offspring. Download Table 7-6, DOCX file.

10.1523/ENEURO.0194-23.2023.t7-7 Table 7-7 p- values from AD comparation between CON-NA vs CON-A, CAF-NA and CAF-A; CON-A vs CAF-NA, CAF-A; and CAF-NA vs CAF-A in the F1 offspring. Download Table 7-7, DOCX file.

10.1523/ENEURO.0194-23.2023.t7-8 Table 7-8 p- values from AD comparation between CON-NA vs CON-A, CAF-NA and CAF-A; CON-A vs CAF-NA, CAF-A; and CAF-NA vs CAF-A in the F2 offspring. Download Table 7-8, DOCX file.

10.1523/ENEURO.0194-23.2023.t7-9 Table 7-9 p- values from AD comparation between CON-NA vs CON-A, CAF-NA and CAF-A; CON-A vs CAF-NA, CAF-A; and CAF-NA vs CAF-A in the F3 offspring. Download Table 7-9, DOCX file.

## Discussion

Prenatal exposure to HED is a risk factor for long-term neuropsychiatric disorders in the offspring, including anhedonia (Grillo and Reagan, 2015), addiction (Camacho et al., 2017; Cruz-Carrillo et al., 2020), depression (A. L. de la Garza et al., 2019; Trujillo-Villarreal et al., 2021), asocial- (Veniaminova et al., 2017; Maldonado-Ruiz et al., 2022), and anxiety-like behaviors (Edlow, 2018). Here, we newly identified that exposure to HED during the prenatal stage primes the transgenerational inheritance of brain structural alterations that code for anxiety-like behavior in the F1, F2, and F3 generations. Our study highlights the structural defects in the hippocampal layer CA3 and amygdala in the F1, F2, and F3 generations exhibiting an anxiety-like behavior. This is the first study confirming the transgenerational inheritance of brain structural alterations in rats exhibiting an anxiety-like behavior dictated by prenatal exposure to HED.

A major contribution of this study proposed prenatal diet exposure as a trigger of the transgenerational inheritance of anxiety-like behavior in the offspring. Initial epidemiological reports confirmed that prenatal programming by undernutrition after the famines occurring during War World II and Overkalix (Susser, 1996); promoted metabolic alterations and susceptibility to cardiac failure and schizophrenia in the F1 descendants (Barker, 1990; Susser, 1996; Reisinger et al., 2015; Van den Bergh et al., 2020). Preclinical studies showed that prenatal exposure to HED is also a risk factor for long-term neuropsychiatric disorders in the offspring (Peleg-Raibstein et al., 2012; Sharma and Fulton, 2013; Sasaki et al., 2014; Winther et al., 2018; Neuhaus et al., 2020). Our data agrees with these reports by showing that the F1 rats exposed to HED prenatally and during lactation developed anxiety-like behavior after birth, evidenced by longer time spent in the edges and less time in the center of the OF test, increased time to reach the pellet during the NSFT, and less time spent in the closed arms during the EM. Notably, anxiety traits found in the F1 rats were transgenerationally inherited by the F2 and F3 generations, displaying increased time to reach the pellet during the NSFT and less time spent in the closed arms during the EM. While some anxiety traits seemed to be lost during the inter- and transgenerational inheritance to the F2 and F3 generations (OF parameters), rats still preserved behavioral alterations resembling the anxiety-like phenotype. In the context of our study design, it is crucial to recognize that our deliberate decision to focus exclusively on the rats with anxiety-like behavior lineage served our primary goal of exploring hereditary influences on behavior. By concentrating on this specific lineage, we were able to examine the extent to which these behaviors persisted across generations. While we acknowledge the limitation of not including F2 and F3 controls, our research goals and design align with this selective approach. This targeted methodology allowed us to shed light on the transgenerational aspects of anxiety-like behaviors and their hereditary components, providing valuable insights into the interplay between maternal diet and offspring behavior.

A second major finding was that transgenerational inheritance of structural brain changes correlates with anxiety-like behavior in the F1, F2, and F3 generations. Structural alterations in the brain have been proposed as a contributor to neuropsychiatric traits in anxiety (McIlwrath et al., 2020), including lower volume in the hippocampus (Cross et al., 2016; Merz et al., 2018), and higher volume in the amygdala (McIlwrath et al., 2020). We used DBM to characterize the effect of diet on local brain volume differences in the F1, F2, and F3 rats exhibiting an anxiety-like behavior. Previously, a study found lower volume within the nucleus accumbens (NAc), hippocampus, and prefrontal cortex of rats exposed to a high-energy diet (HED) during prenatal and lactation periods, and displaying behaviors suggestive of depression (Trujillo-Villarreal et al., 2021). In the current study, we enriched the categorization of diet-dependent structural brain defects and discovered lower brain volume in hippocampal layer CA3 and higher volume in the amygdala of the F1, F2, and F3 rats exhibiting an anxiety-like behavior. Accordingly, clinical studies have documented volumetric alterations in the hippocampus and amygdala associated with a state of anxiety in human patients (Modi et al., 2013; Li et al., 2019; Lee et al., 2021; Zhang et al., 2022). A recent report documented that the F1 generation of mice prenatally and during lactation exposed to HED also exhibited volume changes in the medial amygdala and in the basal forebrain, supporting our findings (Fernandes et al., 2021). Another study showed transgenerational impairment in hippocampal synaptic plasticity and cognitive function were documented in the F1, F2, and F3 generations following maternal exposure to HED (C. Lin et al., 2021). This evidence consistently supports our findings, suggesting that prenatal exposure to HED primes the transgenerational inheritance of volumetric alterations in the hippocampal layer CA3 and amygdala that code for anxiety-like behavior in rodents.

We then established associations between the effect of “diet” on the transgenerational inheritance of “brain structure” and “behavioral outcomes”. We found a significant effect of diet on the transgenerational inheritance of volume changes in right hippocampal layer CA3, cerebellar white matter, and cerebellar lobule 6 shared between CON-NA versus CAF-A individuals of F1 and F3 offspring. The hippocampus, as a part of the limbic system, modulates anxiety (Fasick et al., 2015), and a reduced volume in the amygdala-hippocampus contributes to an anxiety outcome (Baur et al., 2012; Salokangas et al., 2021). Amygdala activation has been shown to be higher in anxious human patients compared with healthy controls (Makovac et al., 2018), and individuals with generalized anxiety disorders have reduced connections between the amygdala and the prefrontal cortex (Yang et al., 2020). Structural volume changes in the cerebellum and their effect on anxiety are less clear. Initial reports showed structural abnormalities in the cerebellum of individuals exposed to heroin and exhibiting depression and anxiety (W. C. Lin et al., 2012). A recent study also documented defective functional connectivity between the cerebellar and cerebral cortex in adolescents with anxiety (Lee et al., 2021). However, no reports have confirmed the transgenerational inheritance of brain volume alterations in the hippocampus or cerebellum coding for anxiety in humans or murine models. Additionally, in the context of our study exploring the transgenerational effects of prenatal diet on anxiety-like behavior, notable local volumetric changes were predominantly observed in the right hemisphere. Specifically, regions such as the amygdala, hippocampus, hypothalamus, and cerebellum exhibited significant alterations in volume. These findings highlight the potential role of specific brain regions, particularly those in the right hemisphere, in mediating the transgenerational effects associated with anxiety-like behavior. Regarding the processing of emotions in the hemispheres, two main theories have been proposed. The first theory suggests hemispheric specialization, where emotional processing occurs primarily in the right hemisphere, regardless of the emotional valence. It is also suggested that initial emotional processing occurs in the right hemisphere before being transferred to the left hemisphere for higher-order appraisal and control (Bruder et al., 2016, Gibson et al., 2022). The second theory proposes that the hemispheres have distinct roles based on the valence of the emotion, with the right hemisphere being more involved in processing negative emotions, while the left hemisphere is more engaged in processing positive emotions (Gibson et al., 2022). These theories provide a framework for understanding the complex interplay between brain lateralization and emotional processing. Overall, our findings highlight the significance of the right hemisphere and specific brain regions, such as the amygdala, hippocampus, hypothalamus, and cerebellum, in the transgenerational effects related to anxiety-like behavior. Here, we propose for the first time that prenatal exposure to HED efficiently primes the transgenerational inheritance of brain regions that code for anxiety-like behavior highlighting the cerebellum, hypothalamus, amygdala, and hippocampus as the major targets of defective behavior.

Another important finding in the present study is the transgenerational inheritance of microstructural brain alteration into the third generation, primed by prenatal exposure to HED. Using DWI we analyzed the ADC, FA (measure of the directionality of diffusion), radial diffusivity (RD, mainly a myelin marker reflecting diffusion perpendicular to axonal tracts), and AD (axonal marker which reflects diffusion along the principal direction of the fiber) as the major parameters during the DWI analysis (Huisman, 2010; Ayling et al., 2012; Meng et al., 2021). We found that the F2 nonanxious individuals born from F1 subjects exposed to CAF diet (CAF-NA) had higher FA in the hippocampus compared with the nonanxious control group (CON-NA). One study in human patients diagnosed with anxiety showed hippocampal volume reductions, as well as reduced FA and mean diffusivity, which the authors interpreted as a potential underlying cause of the significantly increased scores of anxiety and depression (Chen et al., 2019). Accordingly, we found that in contrast to the F3 CON-NA and CAF-NA, the F3 CAF-A offspring displayed higher FA and AD values in the right amygdala, higher AD value in the hippocampus compared with CAF nonanxious subjects, and higher ADC values in the left CC compared with CON-NA. Higher FA in gray matter is associated with decreased complexity of dendritic arbors whereas higher FA in white matter is associated with increased integrity (Juranek et al., 2012). Furthermore, an increase in the FA values alters the amygdala–ventromedial prefrontal cortical pathway in individuals diagnosed with anxiety (Kim and Whalen, 2009). Conversely, a study showed a higher connection between the right medial prefrontal cortex and the CC in anxious individuals evidenced by an increase in the FA values (Ayling et al., 2012). Although white matter deterioration is often associated with lower FA, a potential proposal might suggest that elevated FA values found in the F3 CAF-A offspring may be a sign of neuroinflammation, increased myelination, axonal structural damage, or decreased axonal diameter (Sengul et al., 2019). A recent report confirmed an increase in the structural connectivity between the mPFC and the amygdala of anxious rats exposed to a HED (Vega-Torres et al., 2018). On the other hand, epigenetic changes have also been proposed as a potential mechanism underlying the transgenerational effects of prenatal high-calorie diet exposure. Epigenetic changes, such as DNA methylation and histone modifications, can alter gene expression patterns and have been implicated in the transmission of phenotypic traits across generations (Dunn et al., 2019). In a previous study, it was reported that exposure to a high-calorie diet during prenatal stages resulted in an increase in global DNA methylation within the nucleus accumbens (Cruz-Carrillo et al., 2020). Our findings support the hypothesis that prenatal diet-induced epigenetic modifications play a role in the transgenerational carryover of behavioral and brain alterations. Further research is needed to corroborate this and elucidate the specific epigenetic mechanisms involved and their impact on gene expression and neurodevelopment.

Notably, alterations in dietary fat composition also lead to shifts in milk lipid composition and the daily production of milk lipids in nursing animals (Flint et al., 2005, Mendes-da-Silva et al., 2014). In both contexts, there appears to be a susceptibility in the offspring's brain development to the potential impacts of lipotoxicity or lipid peroxidation induced by maternal high-fat intake. Regrettably, in our study, the individual effects of cafeteria diet consumption during pregnancy and lactation were not isolated; instead, the model represents the combined influence of both phases.

While our study has provided valuable insights into the transgenerational effects of prenatal high-calorie diet exposure on brain structure and anxiety-like behavior, there are several avenues for further investigation. Further research is warranted to unravel the underlying mechanisms linking prenatal high-calorie diet exposure to the observed transgenerational brain alterations and anxiety-like behavior. Also, longitudinal studies following the offspring into adulthood are needed to assess the persistence of the observed brain alterations and behavioral changes over time. Examining whether these effects persist into later stages of life and whether they manifest as clinically significant outcomes could have implications for early intervention and preventive strategies.

There are several limitations to consider in our study that may impact the interpretation and generalizability of our findings. Our study did not investigate sex differences in the transgenerational inheritance of anxiety-like phenotypes. We focused on male offspring to align with the specific research objectives. However, we recognize the significance of studying both sexes and the potential implications of prenatal diet on female rats. In any case, female offspring were allocated to an additional experimental protocol under investigation. Furthermore, we would like to highlight the disparity in litter sizes between groups and within generations. Although we ensured a consistent count of six pups per female during weaning, there was an imbalance in litter sizes among the groups. This discrepancy may introduce variability and potentially confound the interpretation of our results. A notable limitation of our study is the small sample size observed in the control group where the occurrence of anxious behavior was lower. However, this limited sample size also highlights the robustness of our findings in the treatment group, where significant effects were still detected despite the challenging conditions of a small control group. Future studies with larger sample sizes should be conducted to tackle this limitation.

In conclusion, our findings suggest that prenatal exposure to HED programs the transgenerational inheritance of anxiety-like behavior and promotes structural and diffusional brain changes in the offspring. We propose that diet-dependent structural abnormalities in the cerebellum, hypothalamus, amygdala, and hippocampus are preserved up to the F3 generation of anxious individuals. Collectively, our study enriches the comprehension of the role of exposure to HED during gestation and lactation on setting brain structural abnormalities that code for the transgenerational inheritance of anxiety-like phenotypes in the young offspring.

## Synthesis

Reviewing Editor: Julie Bakker, University of Liege Decisions are customarily a result of the Reviewing Editor and the peer reviewers coming together and discussing their recommendations until a consensus is reached. When revisions are invited, a fact-based synthesis statement explaining their decision and outlining what is needed to prepare a revision will be listed below. The following reviewer(s) agreed to reveal their identity: NONE. Note: If this manuscript was transferred from JNeurosci and a decision was made to accept the manuscript without peer review, a brief statement to this effect will instead be what is listed below.

Synthesis: the paper has been reviewed again and both reviewers agree that the comments have been addressed in a satisfactorily manner, but remain rather critical about the general design of the study, see their comments below.

Reviewer 1:

The authors were very responsive to reviews and have made revisions that have clarified the previously raised issues. The authors provided the required methodological information sufficient for an independent assessment of the analysis and data interpretation. The revised findings support the assumptions and model underlying the putative transgenerational effects of hypercaloric diets in brain and behavior measures in male rats. Limitations are clearly described.

Reviewer 2:

As mentioned twice before, this is very incremental to prior work by the group. responses to prior reviews did not address this point and i do not see a path for pursuing it.

For future papers, it is largely considered unacceptable to study only males or only females, even of the other sex is "" subjected to another protocol". if your question is important to ask, then you should ask it in both sexes.
