# Supplementary material for: Paternal Prenatal and Lactation Exposure to a High-Calorie Diet Shapes Transgenerational Brain Macro- and Microstructure Defects, Impacting Anxiety-Like Behavior in Male Offspring Rats
Source: eNeuro. 2024 Feb 9;11(2):ENEURO.0194-23.2023. doi: 10.1523/ENEURO.0194-23.2023 (PMC10863632; doi:10.1523/ENEURO.0194-23.2023)
Supplement: Table 2-1 — Results of Two-Way Analysis of Variance (ANOVA) for diet group and filial group comparison in behavioral variables. Download Table 2-1, DOCX file. [file eneuro-11-ENEURO.0194-23.2023-s008.docx]

Extended Data Table 2-1. Results of Two-Way Analysis of Variance (ANOVA) for diet group and filial group comparison in behavioral variables.

| Model |  | Degree of Freedom | Sum of squares | Mean Square | F-value | Adjusted  p-value | η² |
| --- | --- | --- | --- | --- | --- | --- | --- |
| LFOF ~ group * F | Diet Group | 1 | 520192 | 520192 | 16.168 | 0.0001*** | 0.108 |
|  | Filial | 2 | 331036 | 165518 | 5.145 | 0.0071** | 0.069 |
|  | Diet Group * Filial | 2 | 39162 | 19581 | 0.609 | 0.5457 | 0.008 |
|  | | | | | | |  |
| LFC ~ group * F | Diet Group | 1 | 94768 | 94768 | 6.385 | 0.0126* | 0.042 |
|  | Filial | 2 | 13240 | 6620 | 0.446 | 0.6410 | 0.005 |
|  | Diet Group * Filial | 2 | 9891 | 4945 | 0.333 | 0.7172 | 0.004 |
|  | | | | | | |  |
| FI ~ group * F | Diet Group | 1 | 3.3 | 3.35 | 0.808 | 0.3702 | 0.004 |
|  | Filial | 2 | 196.8 | 98.40 | 23.770 | <0.0001*** | 0.239 |
|  | Diet Group * Filial | 2 | 20.7 | 10.37 | 2.506 | 0.0851 | 0.025 |
|  | | | | | | |  |
| TSOA ~ group * F | Diet Group | 1 | 614 | 613.7 | 2.781 | 0.0976 | 0.017 |
|  | Filial | 2 | 3766 | 1882.9 | 8.533 | 0.0003*** | 0.105 |
|  | Diet Group * Filial | 2 | 742 | 370.9 | 1.681 | 0.1899 | 0.020 |
|  | | | | | | |  |
| TSCA ~ group * F | Diet Group | 1 | 8624 | 8624 | 9.445 | 0.0025** | 0.054 |
|  | Filial | 2 | 21619 | 10809 | 11.839 | <0.0001*** | 0.135 |
|  | Diet Group * Filial | 2 | 101 | 50 | 0.055 | 0.9462 | <0.001 |
|  | | | | | | |  |
| Imm ~ group * F | Diet Group | 1 | 7941 | 7941 | 2.635 | 0.1068 | 0.015 |
|  | Filial | 2 | 40116 | 20058 | 6.656 | 0.0017** | 0.076 |
|  | Diet Group * Filial | 2 | 67036 | 33518 | 11.122 | <0.0001*** | 0.127 |
|  | | | | | | |  |
| Edge ~ group * F | Diet Group | 1 | 114 | 113.9 | 0.442 | 0.5078 | 0.003 |
|  | Filial | 2 | 3945 | 1972.5 | 7.649 | 0.0007*** | 0.121 |
|  | Diet Group * Filial | 2 | 991 | 495.6 | 1.922 | 0.1514 | 0.030 |
|  | | | | | | |  |
| Center ~ group * F | Diet Group | 1 | 142 | 142.1 | 0.549 | 0.4603 | 0.004 |
|  | Filial | 2 | 4167 | 2083.4 | 8.053 | 0.0005*** | 0.128 |
|  | Diet Group * Filial | 2 | 883 | 441.6 | 1.707 | 0.1864 | 0.027 |
|  | | | | | | |  |
| Distance ~ group * F | Diet Group | 1 | 7119 | 7119 | 0.017 | 0.8970 | <0.001 |
|  | Filial | 2 | 77725248 | 38862624 | 92.579 | <0.0001*** | 0.624 |
|  | Diet Group * Filial | 2 | 1467350 | 733675 | 1.748 | 0.1790 | 0.011 |

*Behavioral traits in the offspring of mice prenatally exposed to high-energy diets*. LFOF = Latency to feed in arena of NFSFT; LFC = Latency to feed in homecage after NSFT; TSOA and TSCA = Time spends in open and closed arms of elevated maze; Imm = Time of immobility in forced swimming test; Edge and Center = Time spent in edge and center of the open field; Distance = Distance traveled during open field; * <0.05, ** <0.01, ***<0.001
