# Supplementary figures and images for: Paternal Prenatal and Lactation Exposure to a High-Calorie Diet Shapes Transgenerational Brain Macro- and Microstructure Defects, Impacting Anxiety-Like Behavior in Male Offspring Rats
Source: eNeuro. 2024 Feb 9;11(2):ENEURO.0194-23.2023. doi: 10.1523/ENEURO.0194-23.2023 (PMC10863632; doi:10.1523/ENEURO.0194-23.2023)

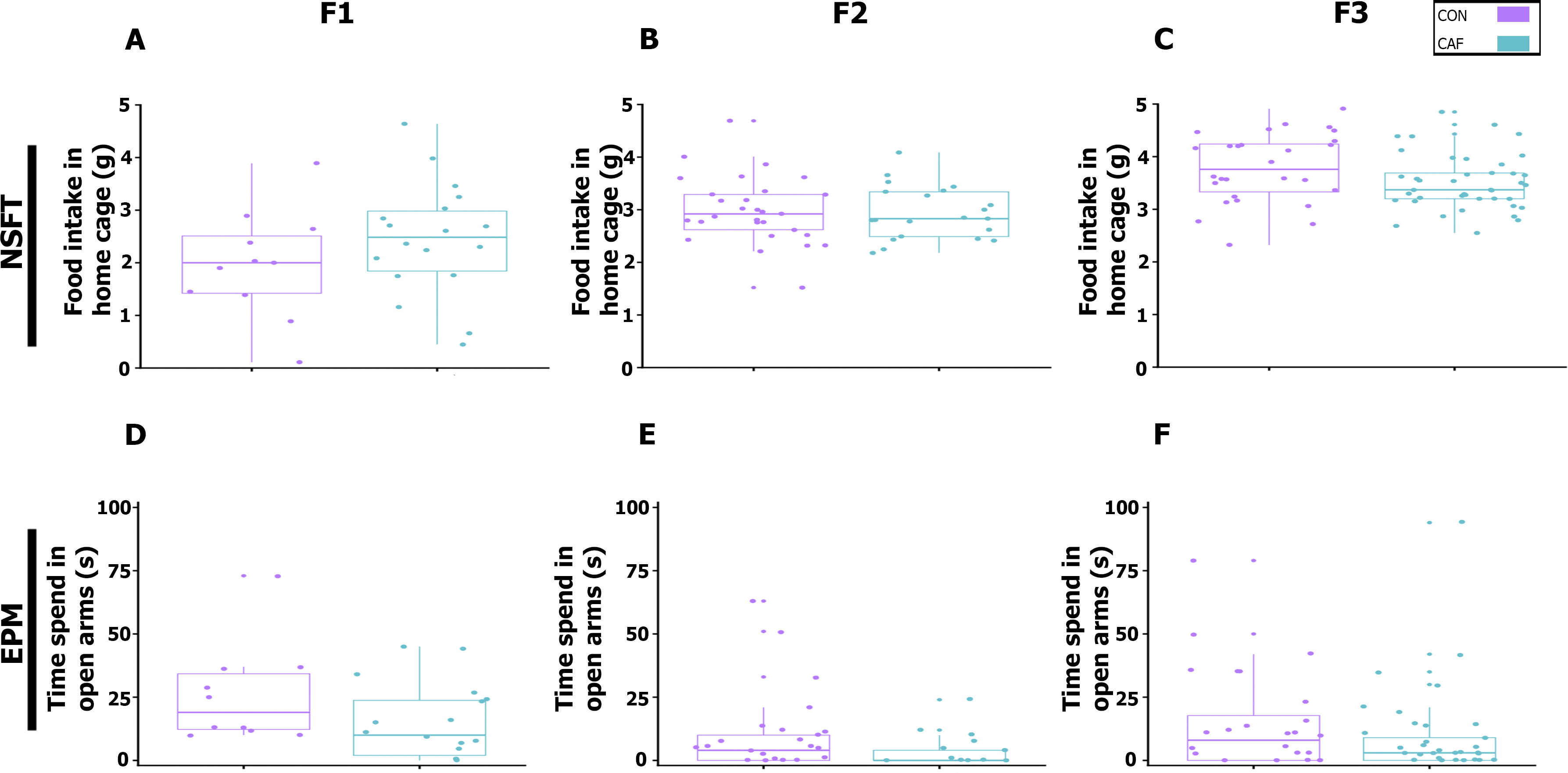

Supplement: Figure 2-1 — Box plots comparing food intake in the home-cage after reaching the pellet in the Novelty Suppressed Feeding Test (NSFT) and time spent in open arms during the Elevated Maze (EM) across groups categorized by F0 diet (CON, CAF) and offspring. Panels A and D show F1, panels B and E show F2, and panels C and F show F3. Significant differences between groups are denoted by asterisks: * p < 0.05, ** p < 0.01, and *** p < 0.001. Specific group differences were determined through post hoc t-test analysis. Download Figure 2-1, TIF file. [file eneuro-11-ENEURO.0194-23.2023-s002.tif]

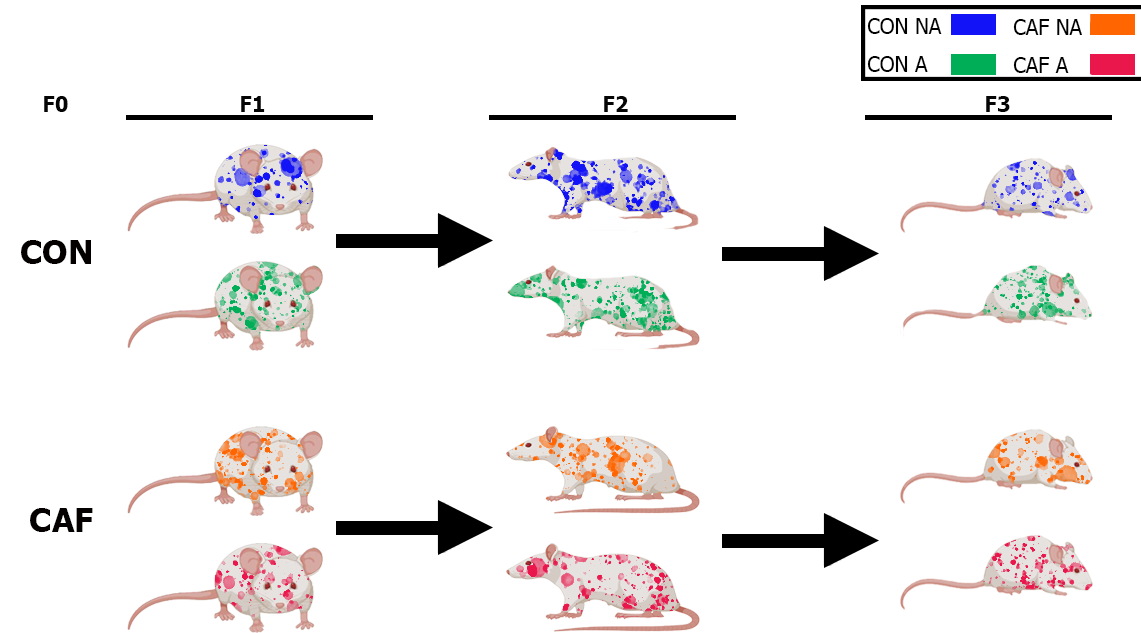

Supplement: Figure 4-1 — Color-coded representation of each behavioral phenotype accordingly to each generation and diet group. Download Figure 4-1, TIF file. [file eneuro-11-ENEURO.0194-23.2023-s003.tif]

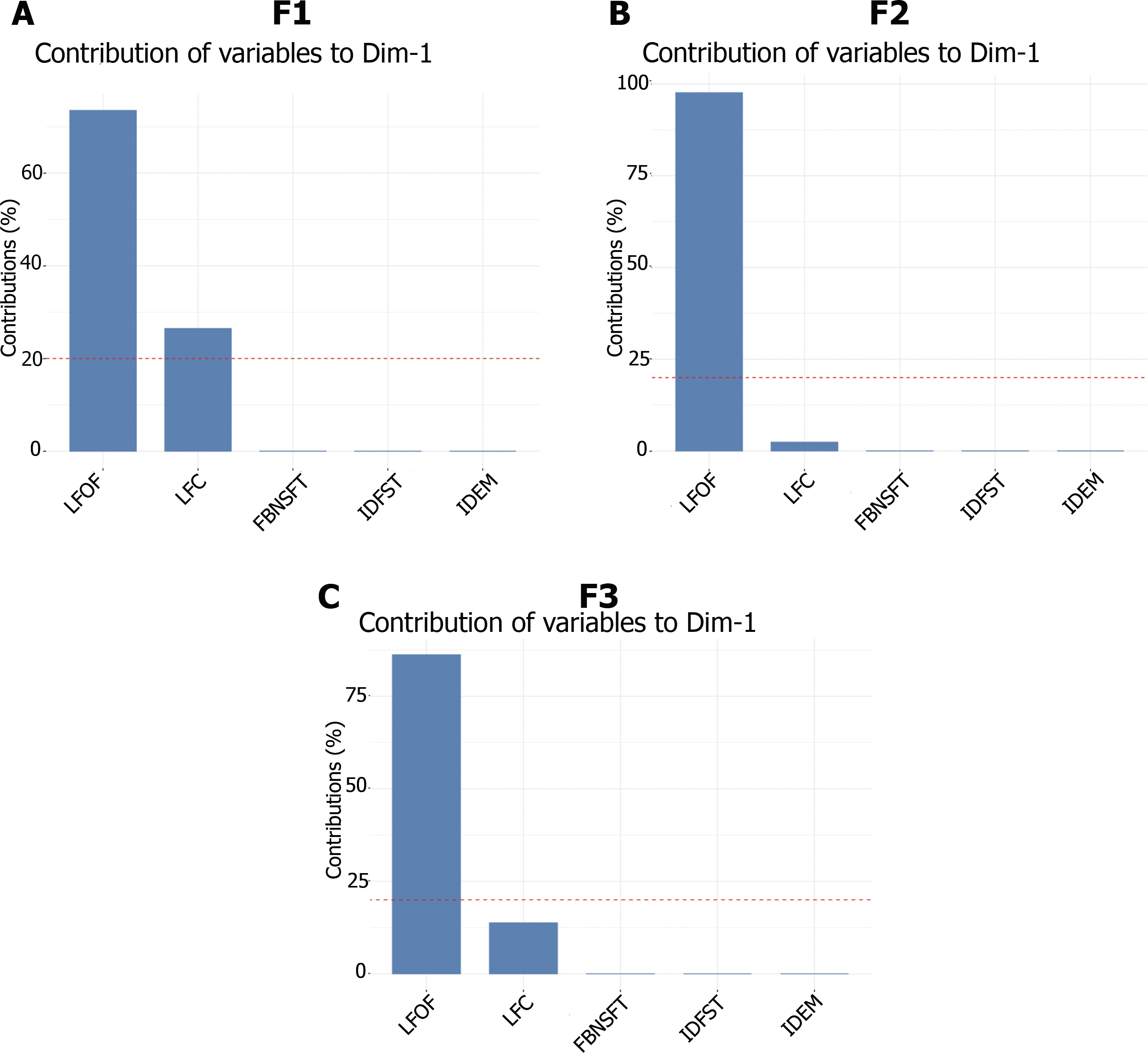

Supplement: Figure 4-2 — PCA contribution of variables to variance in A) F1, B) F2 and C) F3. Download Figure 4-2, TIF file Figure 4-2, DOCX file. [file eneuro-11-ENEURO.0194-23.2023-s004.tif]

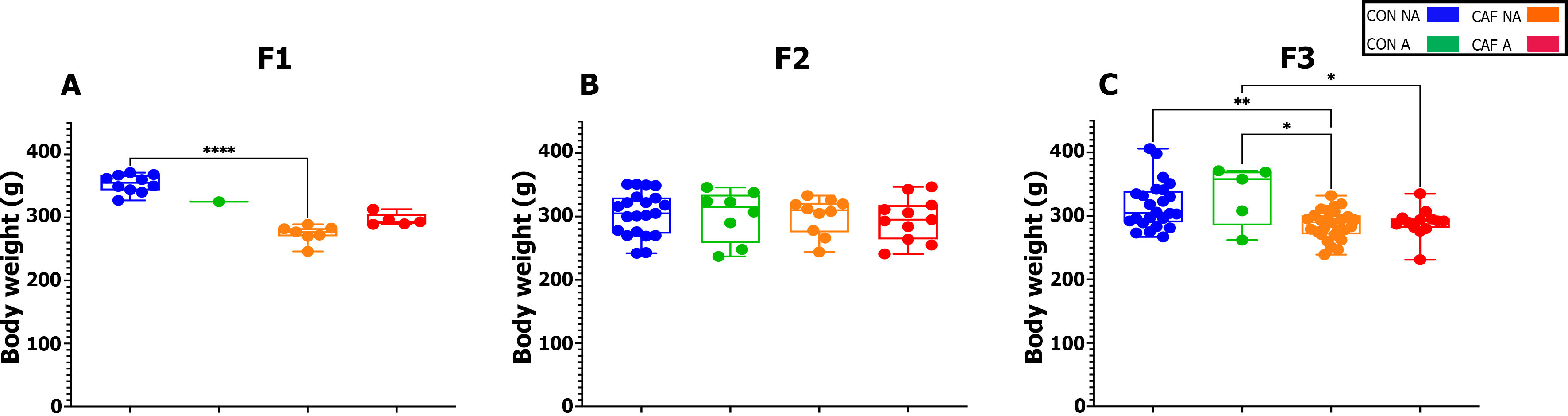

Supplement: Figure 5-1 — Boxplot comparing body weights across groups categorized by F0 diet (CON, CAF), behavioral phenotype, and offspring A) F1, B) F2, and C) F3. Significant differences between groups are indicated by asterisks, with * p < 0.05, ** p < 0.01, and *** p < 0.001. Post hoc Tukey's analysis was utilized to determine specific group differences. Download Figure 5-1, TIF file. [file eneuro-11-ENEURO.0194-23.2023-s005.tif]

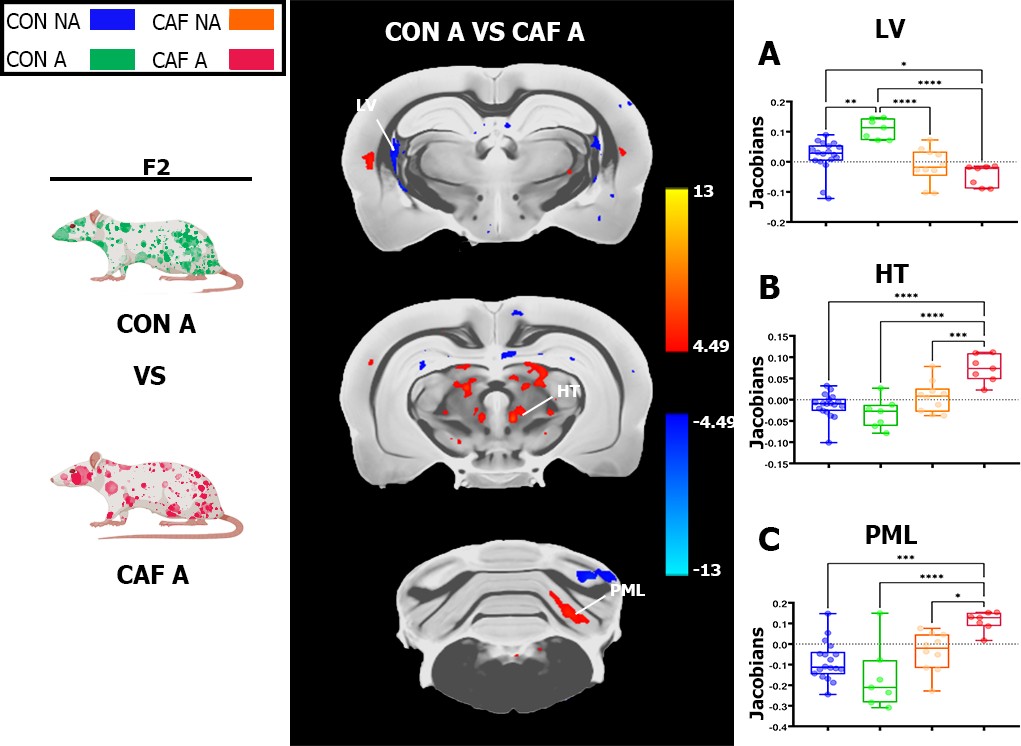

Supplement: Figure 5-2 — Representative coronal MRI section showing affected areas that were found to increase (red) or decrease (blue) among each contrast (CON A vs CAF A) comparation color-coded as the phenotype group alongside the box plot showing volume differences (y axis = Jacobians) in each group (x axis) in specific region with higher significant changes in DBM analysis, whereas F2 Jacobian values was compared in A) LV, B) HT C) PML. Results are expressed as mean + SEM, following by ANOVA post hoc Tukey. *p<0.05. Download Figure 5-2, TIF file. [file eneuro-11-ENEURO.0194-23.2023-s006.tif]
