# Supplementary material for: Paternal Prenatal and Lactation Exposure to a High-Calorie Diet Shapes Transgenerational Brain Macro- and Microstructure Defects, Impacting Anxiety-Like Behavior in Male Offspring Rats
Source: eNeuro. 2024 Feb 9;11(2):ENEURO.0194-23.2023. doi: 10.1523/ENEURO.0194-23.2023 (PMC10863632; doi:10.1523/ENEURO.0194-23.2023)
Supplement: Figure 4-2 — PCA contribution of variables to variance in A) F1, B) F2 and C) F3. Download Figure 4-2, TIF file Figure 4-2, DOCX file. [file eneuro-11-ENEURO.0194-23.2023-s007.docx]

Table 6. Results of Two-Way Analysis of Variance (ANOVA) for prenatal diet and behavioral phenotype group comparison in brain region of F2 corresponding to Extended Data Figure 4-2.

| Region | Model |  | Degree of Freedom | Sum of squares | Mean Square | F-value | Adjusted  p-value |
| --- | --- | --- | --- | --- | --- | --- | --- |
| PML  Contrast  CON-A  Vs  CAF-A | PML ~ PD * phenotype | PD | 1 | 0.0497 | 0.0497 | 19.820 | 0.0001*** |
|  |  | phenotype | 1 | 0.0114 | 0.0114 | 4.541 | 0.0396* |
|  |  | PD * phenotype | 1 | 0.0345 | 0.0345 | 13.787 | 0.0006*** |
| FrA  Contrast  CON-A  Vs  CAF-A | FrA ~ PD * phenotype | PD | 1 | 0.1695 | 0.1695 | 16.343 | 0.0002*** |
|  |  | phenotype | 1 | 0.0048 | 0.0048 | 0.463 | 0.5005 |
|  |  | PD * phenotype | 1 | 0.1168 | 0.1167 | 11.259 | 0.0018** |
| HT  Contrast  CON-A  Vs  CAF-A | HT ~ PD * phenotype | PD | 1 | 0.0280 | 0.0280 | 26.523 | 0.0001*** |
|  |  | phenotype | 1 | 0.0035 | 0.0035 | 3.314 | 0.0765 |
|  |  | PD * phenotype | 1 | 0.0166 | 0.0166 | 15.730 | 0.0003*** |

*Behavioral traits in the offspring of mice prenatally exposed to high-energy diets*. PD = Distance traveled during open field; * <0.05, ** <0.01, ***<0.001
