# Supplementary material for: Paternal Prenatal and Lactation Exposure to a High-Calorie Diet Shapes Transgenerational Brain Macro- and Microstructure Defects, Impacting Anxiety-Like Behavior in Male Offspring Rats
Source: eNeuro. 2024 Feb 9;11(2):ENEURO.0194-23.2023. doi: 10.1523/ENEURO.0194-23.2023 (PMC10863632; doi:10.1523/ENEURO.0194-23.2023)
Supplement: Table 7-1 — p- values from FA comparation between CON-NA vs CON-A, CAF-NA and CAF-A; CON-A vs CAF-NA, CAF-A; and CAF-NA vs CAF-A in the F1 offspring. Download Table 7-1, DOCX file. [file eneuro-11-ENEURO.0194-23.2023-s009.docx]

Extended Data Table 7-1. p- values from FA comparation between CON-NA vs CON-A, CAF-NA and CAF-A; CON-A vs CAF-NA, CAF-A; and CAF-NA vs CAF-A in the F1 offspring.

| Region | ANOVA | CON-NA VS. CON-A | CON-NA VS. CAF-NA | CON-NA VS. CAF-A | CON-A VS. CAF-NA | CON-A VS. CAF-A | CAF-NA VS. CAF-A | Effect size (η) |
| --- | --- | --- | --- | --- | --- | --- | --- | --- |
| Right corpus callosum | F (3, 18) = 0.4498  P=0.7205 | P=0.9268 | P=0.8758 | P=0.9964 | P=0.7282 | P=0.8747 | P=0.914 | 0.069 |
| Left corpus callosum | F (3, 18) = 1.903  P=0.1654 | P=0.8892 | P=0.2561 | P=0.9312 | P=0.3049 | P=0.7167 | P=0.3967 | 0.240 |
| Fornix | F (3, 18) = 0.05926  P=0.9805 | P=0.998 | P=0.9999 | P=0.9912 | P=0.9963 | P=0.9866 | P=0.9955 | 0.009 |
| Right fimbria | F (3, 16) = 0.2750  P=0.8426 | P=>0.9999 | P=0.8948 | P=0.848 | P=0.9792 | P=0.972 | P=0.9999 | 0.049 |
| Left fimbria | F (3, 16) = 0.6956  P=0.5681 | P=>0.9999 | P=0.5567 | P=0.7961 | P=0.8405 | P=0.9438 | P=0.9348 | 0.115 |
| Right internal capsule | F (3, 18) = 0.06697  P=0.9767 | P=0.9728 | P=0.9981 | P=0.9999 | P=0.9862 | P=0.9758 | P=0.9992 | 0.011 |
| Left internal capsule | F (3, 18) = 0.2723  P=0.8446 | P=0.8903 | P=0.9909 | P=0.9988 | P=0.9428 | P=0.8431 | P=0.9579 | 0.312 |
| Cerebelar lobe 3 | F (3, 18) = 0.8419  P=0.4887 | P=0.9848 | P=0.9852 | P=0.7401 | P=0.9455 | P=0.9983 | P=0.4641 | 0.123 |
| Cerebelar lobe 6 | F (3, 18) = 0.08256  P=0.9687 | P=0.9993 | P=0.9823 | P=0.9614 | P=0.9998 | P=0.9992 | P=0.9998 | 0.013 |
| Right hippocampus | F (3, 18) = 0.09111  P=0.9640 | P=>0.9999 | P=0.9842 | P=0.9582 | P=0.9986 | P=0.9964 | P=0.9995 | 0.014 |
| Left hippocampus | F (3, 18) = 0.01158  P=0.9982 | P=>0.9999 | P=0.999 | P=0.999 | P=0.9994 | P=0.9994 | P=>0.999 | 0.001 |
| Right amygdala | F (3, 18) = 0.5352  P=0.6640 | P=0.9876 | P=0.8516 | P=0.9933 | P=0.9994 | P=0.9629 | P=0.6211 | 0.081 |
| Left amygdala | F (3, 15) = 0.5594  P=0.6499 | P=0.999 | P=0.7289 | P=0.9987 | P=0.8682 | P=0.9952 | P=0.7115 | 0.011 |

*p- values from FA analysis in the offspring of mice according to prenatal diet exposure.*
