# Supplementary material for: Paternal Prenatal and Lactation Exposure to a High-Calorie Diet Shapes Transgenerational Brain Macro- and Microstructure Defects, Impacting Anxiety-Like Behavior in Male Offspring Rats
Source: eNeuro. 2024 Feb 9;11(2):ENEURO.0194-23.2023. doi: 10.1523/ENEURO.0194-23.2023 (PMC10863632; doi:10.1523/ENEURO.0194-23.2023)
Supplement: Table 7-2 — p- values from FA comparation between CON-NA vs CON-A, CAF-NA and CAF-A; CON-A vs CAF-NA, CAF-A; and CAF-NA vs CAF-A in the F2 offspring. Download Table 7-2, DOCX file. [file eneuro-11-ENEURO.0194-23.2023-s010.docx]

Extended Data Table 7-2. p- values from FA comparation between CON-NA vs CON-A, CAF-NA and CAF-A; CON-A vs CAF-NA, CAF-A; and CAF-NA vs CAF-A in the F2 offspring

| Region | ANOVA | CON-NA VS. CON-A | CON-NA VS. CAF-NA | CON-NA VS. CAF-A | CON-A VS. CAF-NA | CON-A VS. CAF-A | CAF-NA VS. CAF-A | Effect size (η) |
| --- | --- | --- | --- | --- | --- | --- | --- | --- |
| Right corpus callosum | F (3, 10) = 0.3877  P=0.7644 | P=0.9213 | P=0.973 | P=0.9063 | P=0.8377 | P=0.7639 | P=0.9966 | 0.104 |
| Left corpus callosum | F (3, 10) = 1.515  P=0.2701 | P=0.6416 | P=0.9456 | P=0.4901 | P=0.5073 | P=0.2555 | P=0.8624 | 0.312 |
| Fornix | F (2, 10) = 0.5416  P=0.5980 | NA | P=0.9998 | P=0.5947 | NA | NA | P=0.6945 | 0.097 |
| Right fimbria | F (3, 12) = 0.8706  P=0.4832 | P=0.6183 | P=0.9013 | P=0.985 | P=0.4269 | P=0.8476 | P=0.8279 | 0.178 |
| Left fimbria | F (3, 12) = 0.9616  P=0.4424 | P=0.8009 | P=0.6627 | P=0.9986 | P=0.3916 | P=0.9053 | P=0.7035 | 0.193 |
| Right internal capsule | F (2, 8) = 0.04783  P=0.9536 | NA | P=0.9996 | P=0.9591 | NA | NA | P=0.9607 | 0.011 |
| Left internal capsule | F (2, 8) = 2.144  P=0.1796 | NA | P=0.1889 | P=0.9978 | NA | NA | P=0.2698 | 0.348 |
| Cerebelar lobe 3 | F (3, 10) = 0.7434  P=0.5502 | P=0.9703 | P=0.698 | P=0.7756 | P=0.7183 | P=0.7645 | P=0.9993 | 0.182 |
| Cerebelar lobe 6 | F (3, 10) = 0.5295  P=0.6721 | P=0.9989 | P=0.9931 | P=0.7157 | P=>0.9999 | P=0.8666 | P=0.6827 | 0.137 |
| Right hippocampus | F (3, 12) = 3.793  P=0.0401 | P=0.9123 | P=0.0489* | P=0.5915 | P=0.0695 | P=0.4589 | P=0.5167 | 0.486 |
| Left hippocampus | F (3, 12) = 1.195  P=0.3532 | P=0.997 | P=0.521 | P=0.5361 | P=0.6307 | P=0.6422 | P=>0.9999 | 0.230 |
| Right amygdala | F (3, 6) = 1.307  P=0.3558 | P=0.7266 | P=0.8038 | P=0.652 | P=0.4623 | P=0.3743 | P=0.9945 | 0.395 |
| Left amygdala | F (3, 4) = 4.183  P=0.1003 | P=0.1673 | P=0.3782 | P=0.993 | P=0.0802 | P=0.3624 | P=0.4611 | 0.758 |

*p- values from FA analysis in the offspring of mice according to prenatal diet exposure.*
