# Supplementary material for: Paternal Prenatal and Lactation Exposure to a High-Calorie Diet Shapes Transgenerational Brain Macro- and Microstructure Defects, Impacting Anxiety-Like Behavior in Male Offspring Rats
Source: eNeuro. 2024 Feb 9;11(2):ENEURO.0194-23.2023. doi: 10.1523/ENEURO.0194-23.2023 (PMC10863632; doi:10.1523/ENEURO.0194-23.2023)
Supplement: Table 7-3 — p- values from FA comparation between CON-NA vs CON-A, CAF-NA and CAF-A; CON-A vs CAF-NA, CAF-A; and CAF-NA vs CAF-A in the F3 offspring. Download Table 7-3, DOCX file. [file eneuro-11-ENEURO.0194-23.2023-s011.docx]

Extended Data Table 7-3. p- values from FA comparation between CON-NA vs CON-A, CAF-NA and CAF-A; CON-A vs CAF-NA, CAF-A; and CAF-NA vs CAF-A in the F3 offspring

| Region | ANOVA | CON-NA VS. CON-A | CON-NA VS. CAF-NA | CON-NA VS. CAF-A | CON-A VS. CAF-NA | CON-A VS. CAF-A | CAF-NA VS. CAF-A | Effect size (η) |
| --- | --- | --- | --- | --- | --- | --- | --- | --- |
| Right corpus callosum | F(3, 27) = 1.171  P=0.3392 | P=0.5791 | P=0.9995 | P=0.7811 | P=0.6249 | P=0.2672 | P=0.7273 | 0.115 |
| Left corpus callosum | F (3, 27) = 0.9746  P=0.4192 | P=0.8114 | P=0.9729 | P=0.7465 | P=0.9292 | P=0.4285 | P=0.5295 | 0.097 |
| Fornix | F (3, 26) = 0.1673  P=0.9174 | P=0.9111 | P=>0.9999 | P=0.9998 | P=0.9127 | P=0.9075 | P=0.9998 | 0.018 |
| Right fimbria | F (3, 26) = 1.333  P=0.2850 | P=0.9253 | P=0.1975 | P=0.7684 | P=0.9457 | P=0.6547 | P=0.0544 | 0.133 |
| Left fimbria | F (3, 26) = 0.8595  P=0.4744 | P=0.956 | P=0.99 | P=0.8225 | P=0.9877 | P=>0.9999 | P=0.9456 | 0.090 |
| Right internal capsule | F (3, 15) = 1.555  P=0.2417 | P=0.4652 | P=0.8241 | P=0.8472 | P=0.7768 | P=0.2437 | P=0.4414 | 0.237 |
| Left internal capsule | F (3, 11) = 1.198  P=0.3556 | P=0.637 | P=0.2943 | P=0.7079 | P=0.9848 | P=0.9949 | P=0.8842 | 0.246 |
| Cerebelar lobe 3 | F (3, 27) = 0.8524  P=0.4775 | P=0.8915 | P=0.7115 | P=0.9189 | P=0.5036 | P=0.6846 | P=0.9937 | 0.086 |
| Cerebelar lobe 6 | F (3, 27) = 0.2625  P=0.8518 | P=0.9711 | P=0.9953 | P=0.9284 | P=0.9331 | P=0.8341 | P=0.9761 | 0.028 |
| Right hippocampus | F (3, 26) = 1.478  P=0.2437 | P=0.7951 | P=0.9764 | P=0.3785 | P=0.6757 | P=0.2765 | P=0.6427 | 0.145 |
| Left hippocampus | F (3, 26) = 1.502  P=0.2374 | P=0.8624 | P=0.7477 | P=0.3686 | P=0.5528 | P=0.3323 | P=0.9041 | 0.147 |
| Right amygdala | F (3, 22) = 6.983  P=0.0018 | P=0.8859 | P=0.9924 | P=0.0028** | P=0.8169 | P=0.0181* | P=0.0076** | 0.487 |
| Left amygdala | F (3, 15) = 0.9691  P=0.4331 | P=0.9961 | P=0.9619 | P=0.8396 | P=0.908 | P=0.9639 | P=0.3622 | 0.162 |

*p- values from FA analysis in the offspring of mice according to prenatal diet exposure.*
