# Supplementary material for: Paternal Prenatal and Lactation Exposure to a High-Calorie Diet Shapes Transgenerational Brain Macro- and Microstructure Defects, Impacting Anxiety-Like Behavior in Male Offspring Rats
Source: eNeuro. 2024 Feb 9;11(2):ENEURO.0194-23.2023. doi: 10.1523/ENEURO.0194-23.2023 (PMC10863632; doi:10.1523/ENEURO.0194-23.2023)
Supplement: Table 7-4 — p- values from ADC comparation between CON-NA vs CON-A, CAF-NA and CAF-A; CON-A vs CAF-NA, CAF-A; and CAF-NA vs CAF-A in the F1 offspring. Download Table 7-4, DOCX file. [file eneuro-11-ENEURO.0194-23.2023-s012.docx]

Extended Data Table 7-4. p- values from ADC comparation between CON-NA vs CON-A, CAF-NA and CAF-A; CON-A vs CAF-NA, CAF-A; and CAF-NA vs CAF-A in the F1 offspring

| Region | ANOVA | CON-NA VS. CON-A | CON-NA VS. CAF-NA | CON-NA VS. CAF-A | CON-A VS. CAF-NA | CON-A VS. CAF-A | CAF-NA VS. CAF-A | Effect size (η) |
| --- | --- | --- | --- | --- | --- | --- | --- | --- |
| Right corpus callosum | F (3, 18) = 2.832  P=0.0675 | P=0.2964 | P=0.6912 | P=0.4806 | P=0.1001 | P=0.0687 | P=0.9941 | 0.310 |
| Left corpus callosum | F (3, 18) = 2.435  P=0.0982 | P=0.4991 | P=0.5233 | P=0.4422 | P=0.1541 | P=0.1377 | P=>0.9999 | 0.288 |
| Fornix | F (3, 18) = 1.356  P=0.2878 | P=0.5759 | P=0.7085 | P=0.993 | P=0.2553 | P=0.4604 | P=0.7644 | 0.184 |
| Right fimbria | F (3, 16) = 0.5811  P=0.6359 | P=0.9107 | P=0.9962 | P=0.8559 | P=0.8474 | P=0.664 | P=0.9181 | 0.098 |
| Left fimbria | F (3, 16) = 0.5181  P=0.6758 | P=0.8334 | P=0.9787 | P=0.9567 | P=0.6904 | P=0.6465 | P=0.9997 | 0.088 |
| Right internal capsule | F (3, 18) = 0.8766  P=0.4716 | P=0.9304 | P=0.7853 | P=0.6609 | P=0.6732 | P=0.6156 | P=0.9992 | 0.127 |
| Left internal capsule | F (3, 18) = 1.345  P=0.2912 | P=0.5381 | P=0.9122 | P=0.8101 | P=0.3292 | P=0.2683 | P=0.998 | 0.183 |
| Cerebelar lobe 3 | F (3, 18) = 0.6799  P=0.5757 | P=0.8082 | P=0.8844 | P=0.9448 | P=0.5658 | P=0.6217 | P=0.993 | 0.101 |
| Cerebelar lobe 6 | F (3, 18) = 0.3956  P=0.7577 | P=0.998 | P=0.7482 | P=0.9948 | P=0.7172 | P=0.9824 | P=0.834 | 0.061 |
| Right hippocampus | F (3, 18) = 0.6822  P=0.5744 | P=0.7798 | P=0.9113 | P=0.9517 | P=0.5565 | P=0.5957 | P=0.9964 | 0.102 |
| Left hippocampus | F (3, 18) = 0.7137  P=0.5565 | P=0.7144 | P=0.9248 | P=0.9892 | P=0.4996 | P=0.5897 | P=0.9784 | 0.106 |
| Right amygdala | F (3, 18) = 0.5514  P=0.6537 | P=0.8588 | P=0.8919 | P=0.973 | P=0.6362 | P=0.725 | P=0.9809 | 0.084 |
| Left amygdala | F (3, 15) = 0.5833  P=0.6351 | P=0.7766 | P=0.9988 | P=0.9569 | P=0.7095 | P=0.5787 | P=0.978 | 0.538 |

*p- values from ADC analysis in the offspring of mice according to prenatal diet exposure.*
