# Supplementary material for: Paternal Prenatal and Lactation Exposure to a High-Calorie Diet Shapes Transgenerational Brain Macro- and Microstructure Defects, Impacting Anxiety-Like Behavior in Male Offspring Rats
Source: eNeuro. 2024 Feb 9;11(2):ENEURO.0194-23.2023. doi: 10.1523/ENEURO.0194-23.2023 (PMC10863632; doi:10.1523/ENEURO.0194-23.2023)
Supplement: Table 7-5 — p- values from ADC comparation between CON-NA vs CON-A, CAF-NA and CAF-A; CON-A vs CAF-NA, CAF-A; and CAF-NA vs CAF-A in the F2 offspring. Download Table 7-5, DOCX file. [file eneuro-11-ENEURO.0194-23.2023-s013.docx]

Extended Data Table 7-5. p- values from ADC comparation between CON-NA vs CON-A, CAF-NA and CAF-A; CON-A vs CAF-NA, CAF-A; and CAF-NA vs CAF-A in the F2 offspring

| Region | ANOVA | CON-NA VS. CON-A | CON-NA VS. CAF-NA | CON-NA VS. CAF-A | CON-A VS. CAF-NA | CON-A VS. CAF-A | CAF-NA VS. CAF-A | Effect size (η) |
| --- | --- | --- | --- | --- | --- | --- | --- | --- |
| Right corpus callosum | F (3, 10) = 0.6188  P=0.6186 | P=0.8452 | P=0.8447 | P=0.6702 | P=0.9929 | P=0.9999 | P=0.9919 | 0.156 |
| Left corpus callosum | F (3, 10) = 0.7098  P=0.5680 | P=0.9125 | P=0.6276 | P=0.7186 | P=0.9992 | P=>0.9999 | P=0.9991 | 0.175 |
| Fornix | F (2, 10) = 0.1434  P=0.8682 | NA | P=0.9373 | P=0.8754 | NA | NA | P=0.9911 | 0.027 |
| Right fimbria | F (3, 12) = 0.3949  P=0.7590 | P=0.8097 | P=0.985 | P=0.9984 | P=0.7365 | P=0.8093 | P=0.9986 | 0.089 |
| Left fimbria | F (3, 12) = 0.4521  P=0.7205 | P=>0.9999 | P=0.9827 | P=0.6723 | P=0.9962 | P=0.8564 | P=0.9155 | 0.101 |
| Right internal capsule | F (2, 8) = 0.9342  P=0.4319 | NA | P=0.7749 | P=0.4081 | NA | NA | P=0.8286 | 0.189 |
| Left internal capsule | F (2, 8) = 0.3038  P=0.7461 | NA | P=0.9153 | P=0.7309 | NA | NA | P=0.9429 | 0.070 |
| Cerebelar lobe 3 | F (3, 10) = 0.3258  P=0.8068 | P=0.8658 | P=0.9066 | P=0.9315 | P=0.9886 | P=0.9833 | P=>0.9999 | 0.089 |
| Cerebelar lobe 6 | F (3, 10) = 1.763  P=0.2176 | P=0.892 | P=0.2049 | P=>0.9999 | P=0.9215 | P=0.8998 | P=0.3048 | 0.345 |
| Right hippocampus | F (3, 12) = 0.6071  P=0.6230 | P=0.7728 | P=0.9223 | P=0.9812 | P=0.5823 | P=0.9433 | P=0.8402 | 0.131 |
| Left hippocampus | F (3, 12) = 0.1670  P=0.9166 | P=0.9033 | P=0.9843 | P=0.9979 | P=0.9874 | P=0.967 | P=0.9988 | 0.040 |
| Right amygdala | F (3, 6) = 0.1152  P=0.9480 | P=0.9782 | P=0.9936 | P=0.9936 | P=0.9506 | P=0.9506 | P=>0.9999 | 0.054 |
| Left amygdala | F (3, 4) = 0.1911  P=0.8974 | P=0.8918 | P=0.9785 | P=0.9999 | P=0.9939 | P=0.9564 | P=0.9939 | 0.125 |

*p- values from ADC analysis in the offspring of mice according to prenatal diet exposure.*
