# Supplementary material for: Paternal Prenatal and Lactation Exposure to a High-Calorie Diet Shapes Transgenerational Brain Macro- and Microstructure Defects, Impacting Anxiety-Like Behavior in Male Offspring Rats
Source: eNeuro. 2024 Feb 9;11(2):ENEURO.0194-23.2023. doi: 10.1523/ENEURO.0194-23.2023 (PMC10863632; doi:10.1523/ENEURO.0194-23.2023)
Supplement: Table 7-6 — p- values from ADC comparation between CON-NA vs CON-A, CAF-NA and CAF-A; CON-A vs CAF-NA, CAF-A; and CAF-NA vs CAF-A in the F3 offspring. Download Table 7-6, DOCX file. [file eneuro-11-ENEURO.0194-23.2023-s014.docx]

Extended Data Table 7-6. p- values from ADC comparation between CON-NA vs CON-A, CAF-NA and CAF-A; CON-A vs CAF-NA, CAF-A; and CAF-NA vs CAF-A in the F3 offspring

| Region | ANOVA | CON-NA VS. CON-A | CON-NA VS. CAF-NA | CON-NA VS. CAF-A | CON-A VS. CAF-NA | CON-A VS. CAF-A | CAF-NA VS. CAF-A | Effect size (η) |
| --- | --- | --- | --- | --- | --- | --- | --- | --- |
| Right corpus callosum | F (3, 27) = 1.736  P=0.1832 | P=0.4378 | P=>0.9999 | P=0.3541 | P=0.4363 | P=0.9971 | P=0.3524 | 0.161 |
| Left corpus callosum | F (3, 27) = 3.011  P=0.0475 | P=0.9997 | P=0.8029 | P=0.0341* | P=0.9589 | P=0.2126 | P=0.1634 | 0.250 |
| Fornix | F (3, 26) = 0.7352  P=0.5405 | P=0.9945 | P=0.5113 | P=0.999 | P=0.9515 | P=0.9989 | P=0.7311 | 0.078 |
| Right fimbria | F (3, 26) = 0.6577  P=0.5854 | P=0.648 | P=0.9989 | P=0.9302 | P=0.5906 | P=0.9305 | P=0.8897 | 0.070 |
| Left fimbria | F (3, 26) = 1.712  P=0.1892 | P=0.3142 | P=0.8564 | P=0.2917 | P=0.6071 | P=0.9954 | P=0.6435 | 0.164 |
| Right internal capsule | F (3, 15) = 1.686  P=0.2127 | P=0.4816 | P=0.6898 | P=0.2119 | P=0.8732 | P=0.9917 | P=0.5986 | 0.252 |
| Left internal capsule | F (3, 12) = 1.552  F (3, 12) = 1.552 | P=0.5372 | P=0.8316 | P=0.2522 | P=0.8239 | P=0.9794 | P=0.4517 | 0.279 |
| Cerebelar lobe 3 | F (3, 27) = 0.3319  P=0.8024 | P=0.9898 | P=0.7573 | P=0.9913 | P=0.9861 | P=0.9999 | P=0.9482 | 0.035 |
| Cerebelar lobe 6 | F (3, 27) = 0.7184  P=0.5497 | P=0.892 | P=0.9348 | P=0.8802 | P=0.6951 | P=0.9993 | P=0.6114 | 0.073 |
| Right hippocampus | F (3, 26) = 1.951  P=0.1462 | P=0.6399 | P=0.4511 | P=0.9504 | P=0.2112 | P=0.8466 | P=0.2887 | 0.180 |
| Left hippocampus | F (3, 26) = 2.011  P=0.1370 | P=0.7817 | P=0.414 | P=0.879 | P=0.2932 | P=0.9673 | P=0.1887 | 0.187 |
| Right amygdala | F (3, 22) = 0.3700  P=0.7754 | P=0.9204 | P=0.9961 | P=0.9045 | P=0.8746 | P=0.9982 | P=0.8337 | 0.050 |
| Left amygdala | F (3, 15) = 0.1591  P=0.9222 | P=>0.9999 | P=0.9487 | P=0.9749 | P=0.9531 | P=0.9745 | P=0.9992 | 0.031 |

*p- values from ADC analysis in the offspring of mice according to prenatal diet exposure.*
