# Supplementary material for: Paternal Prenatal and Lactation Exposure to a High-Calorie Diet Shapes Transgenerational Brain Macro- and Microstructure Defects, Impacting Anxiety-Like Behavior in Male Offspring Rats
Source: eNeuro. 2024 Feb 9;11(2):ENEURO.0194-23.2023. doi: 10.1523/ENEURO.0194-23.2023 (PMC10863632; doi:10.1523/ENEURO.0194-23.2023)
Supplement: Table 7-7 — p- values from AD comparation between CON-NA vs CON-A, CAF-NA and CAF-A; CON-A vs CAF-NA, CAF-A; and CAF-NA vs CAF-A in the F1 offspring. Download Table 7-7, DOCX file. [file eneuro-11-ENEURO.0194-23.2023-s015.docx]

Extended Data Table 7-7. p- values from AD comparation between CON-NA vs CON-A, CAF-NA and CAF-A; CON-A vs CAF-NA, CAF-A; and CAF-NA vs CAF-A in the F1 offspring

| Region | ANOVA | CON-NA VS. CON-A | CON-NA VS. CAF-NA | CON-NA VS. CAF-A | CON-A VS. CAF-NA | CON-A VS. CAF-A | CAF-NA VS. CAF-A | Effect size (η) |
| --- | --- | --- | --- | --- | --- | --- | --- | --- |
| Right corpus callosum | F (3, 18) = 1.784  P=0.1863 | P=0.5221 | P=0.8573 | P=0.5995 | P=0.2827 | P=0.1884 | P=0.9766 | 0.229 |
| Left corpus callosum | F (3, 18) = 1.450  P=0.2614 | P=0.6167 | P=0.9698 | P=0.6494 | P=0.4597 | P=0.2635 | P=0.8858 | 0.194 |
| Fornix | F (3, 18) = 2.261  P=0.1161 | P=0.3363 | P=0.5678 | P=0.9964 | P=0.0951 | P=0.2538 | P=0.5688 | 0.273 |
| Right fimbria | F (3, 16) = 0.7392  P=0.5440 | P=0.6503 | P=0.987 | P=0.9913 | P=0.7412 | P=0.5108 | P=0.8817 | 0.121 |
| Left fimbria | F (3, 16) = 0.4782  P=0.7019 | P=0.6887 | P=0.9768 | P=0.9997 | P=0.8012 | P=0.6789 | P=0.9785 | 0.082 |
| Right internal capsule | F (3, 18) = 1.899  P=0.1660 | P=0.903 | P=0.3065 | P=0.4095 | P=0.3532 | P=0.4354 | P=0.9738 | 0.240 |
| Left internal capsule | F (3, 18) = 2.186  P=0.1250 | P=0.5138 | P=0.4379 | P=0.6995 | P=0.1391 | P=0.2124 | P=0.9162 | 0.267 |
| Cerebelar lobe 3 | F (3, 18) = 0.9121  P=0.4547 | P=0.7629 | P=0.9097 | P=0.8108 | P=0.5362 | P=0.4654 | P=0.9983 | 0.131 |
| Cerebelar lobe 6 | F (3, 18) = 0.4867  P=0.6958 | P=0.7971 | P=0.9786 | P=0.9853 | P=0.6651 | P=0.6726 | P=0.9997 | 0.074 |
| Right hippocampus | F (3, 18) = 0.5755  P=0.6385 | P=0.8358 | P=0.9381 | P=.9354 | P=0.6511 | P=0.6464 | P=>0.9999 | 0.087 |
| Left hippocampus | F (3, 18) = 0.5351  P=0.6641 | P=0.8014 | P=0.9517 | P=0.9834 | P=0.6259 | P=0.673 | P=0.9953 | 0.081 |
| Right amygdala | F (3, 18) = 1.090  P=0.3788 | P=0.7827 | P=0.6425 | P=0.9582 | P=0.3922 | P=0.6075 | P=0.8239 | 0.153 |
| Left amygdala | F (3, 14) = 0.1957  P=0.8976 | P=0.999 | P=0.9708 | P=0.8738 | P=0.9994 | P=0.9905 | P=0.9859 | 0.050 |
