# Supplementary material for: Paternal Prenatal and Lactation Exposure to a High-Calorie Diet Shapes Transgenerational Brain Macro- and Microstructure Defects, Impacting Anxiety-Like Behavior in Male Offspring Rats
Source: eNeuro. 2024 Feb 9;11(2):ENEURO.0194-23.2023. doi: 10.1523/ENEURO.0194-23.2023 (PMC10863632; doi:10.1523/ENEURO.0194-23.2023)
Supplement: Table 7-8 — p- values from AD comparation between CON-NA vs CON-A, CAF-NA and CAF-A; CON-A vs CAF-NA, CAF-A; and CAF-NA vs CAF-A in the F2 offspring. Download Table 7-8, DOCX file. [file eneuro-11-ENEURO.0194-23.2023-s016.docx]

Extended Data Table 7-8. p- values from AD comparation between CON-NA vs CON-A, CAF-NA and CAF-A; CON-A vs CAF-NA, CAF-A; and CAF-NA vs CAF-A in the F2 offspring

| Region | ANOVA | CON-NA VS. CON-A | CON-NA VS. CAF-NA | CON-NA VS. CAF-A | CON-A VS. CAF-NA | CON-A VS. CAF-A | CAF-NA VS. CAF-A | Effect size (η) |
| --- | --- | --- | --- | --- | --- | --- | --- | --- |
| Right corpus callosum | F (3, 10) = 0.6476  P=0.6021 | P=0.9996 | P=0.8084 | P=0.6385 | P=0.9203 | P=0.8464 | P=0.9933 | 0.162 |
| Left corpus callosum | F (3, 10) = 1.614  P=0.2475 | P=0.9004 | P=0.5938 | P=0.3805 | P=0.5268 | P=0.4011 | P=0.9857 | 0.326 |
| Fornix | F (2, 10) = 0.2304  P=0.7983 | NA | P=0.9749 | P=0.7808 | NA | NA | P=0.9195 | 0.044 |
| Right fimbria | F (3, 12) = 0.5454  P=0.6605 | P=0.9562 | P=0.8866 | P=0.9035 | P=0.7669 | P=0.9999 | P=0.6613 | 0.12 |
| Left fimbria | F (3, 12) = 1.885  P=0.1860 | P=0.5949 | P=0.7448 | P=0.5215 | P=0.2964 | P=>0.9999 | P=0.2401 | 0.320 |
| Right internal capsule | F (2, 8) = 1.026  P=0.4011 | NA | P=0.6642 | P=0.3966 | NA | NA | P=0.9002 | 0.204 |
| Left internal capsule | F (2, 8) = 1.007  P=0.4074 | NA | P=0.3859 | P=0.7452 | NA | NA | P=0.8315 | 0.201 |
| Cerebelar lobe 3 | F (3, 10) = 0.8542  P=0.4957 | P=0.9859 | P=0.561 | P=0.6191 | P=0.9602 | P=0.9726 | P=0.9998 | 0.203 |
| Cerebelar lobe 6 | F (3, 10) = 0.8467  P=0.4993 | P=0.9769 | P=0.4255 | P=0.9569 | P=0.9362 | P=0.9999 | P=0.7947 | 0.202 |
| Right hippocampus | F (3, 12) = 1.112  P=0.3827 | P=0.9998 | P=0.5164 | P=0.5164 | P=0.7667 | P=0.7667 | P=>0.9999 | 0.217 |
| Left hippocampus | F (3, 12) = 2.791  P=0.0860 | P=0.701 | P=0.092 | P=0.2856 | P=0.7575 | P=0.9683 | P=0.9288 | 0.410 |
| Right amygdala | F (3, 6) = 0.3676  P=0.7794 | P=0.855 | P=0.9979 | P=0.9616 | P=0.931 | P=0.7369 | P=0.945 | 0.155 |
| Left amygdala | F (3, 3) = 0.6944  P=0.6142 | P=0.8402 | P=0.8402 | P=0.9287 | P=0.6167 | P=0.9972 | P=0.7045 | 0.409 |

*p- values from AD analysis in the offspring of mice according to prenatal diet exposure.*
