# Supplementary material for: Paternal Prenatal and Lactation Exposure to a High-Calorie Diet Shapes Transgenerational Brain Macro- and Microstructure Defects, Impacting Anxiety-Like Behavior in Male Offspring Rats
Source: eNeuro. 2024 Feb 9;11(2):ENEURO.0194-23.2023. doi: 10.1523/ENEURO.0194-23.2023 (PMC10863632; doi:10.1523/ENEURO.0194-23.2023)
Supplement: Table 7-9 — p- values from AD comparation between CON-NA vs CON-A, CAF-NA and CAF-A; CON-A vs CAF-NA, CAF-A; and CAF-NA vs CAF-A in the F3 offspring. Download Table 7-9, DOCX file. [file eneuro-11-ENEURO.0194-23.2023-s017.docx]

Extended Data Table 7-9. p- values from AD comparation between CON-NA vs CON-A, CAF-NA and CAF-A; CON-A vs CAF-NA, CAF-A; and CAF-NA vs CAF-A in the F3 offspring

| Region | ANOVA | CON-NA VS. CON-A | CON-NA VS. CAF-NA | CON-NA VS. CAF-A | CON-A VS. CAF-NA | CON-A VS. CAF-A | CAF-NA VS. CAF-A | Effect size (η) |
| --- | --- | --- | --- | --- | --- | --- | --- | --- |
| Right corpus callosum | F(3, 27) = 1.852  P=0.1616 | P=0.9988 | P=0.9994 | P=0.1719 | P=0.9958 | P=0.3631 | P=0.2049 | 0.170 |
| Left corpus callosum | F (3, 27) = 3.120  P=0.0425 | P=0.9097 | P=0.9823 | P=0.0601 | P=0.8017 | P=0.0802 | P=0.1139 | 0.257 |
| Fornix | F (3, 26) = 0.7505  P=0.5320 | P=0.9871 | P=0.5169 | P=0.9999 | P=0.9711 | P=0.9931 | P=0.6943 | 0.079 |
| Right fimbria | F (3, 26) = 0.8376  P=0.4855 | P=0.8873 | P=0.8357 | P=0.9397 | P=0.5888 | P=0.996 | P=0.6114 | 0.088 |
| Left fimbria | F (3, 26) = 1.658  P=0.2004 | P=0.4406 | P=>0.9999 | P=0.3714 | P=0.4534 | P=0.999 | P=0.3857 | 0.160 |
| Right internal capsule | F (3, 15) = 2.550  P=0.0947 | P=0.9947 | P=0.9714 | P=0.0796 | P=>0.9999 | P=0.3026 | P=0.1198 | 0.337 |
| Left internal capsule | F (3, 12) = 1.665  P=0.2270 | P=0.6642 | P=0.9915 | P=0.3146 | P=0.7021 | P=0.9653 | P=0.2785 | 0.293 |
| Cerebelar lobe 3 | F (3, 27) = 0.1141  P=0.9511 | P=0.9634 | P=0.9769 | P=>0.9999 | P=0.9966 | P=0.9754 | P=0.9898 | 0.012 |
| Cerebelar lobe 6 | F (3, 27) = 0.6021  P=0.6193 | P=>0.9999 | P=0.9976 | P=0.6767 | P=0.9979 | P=0.8732 | P=0.5809 | 0.062 |
| Right hippocampus | F (3, 27) = 2.808  P=0.0585 | P=0.9388 | P=0.2802 | P=0.6484 | P=0.4063 | P=0.9988 | P=0.0512 | 0.238 |
| Left hippocampus | F (3, 26) = 3.806  P=0.0219 | P=0.9043 | P=0.5508 | P=0.1324 | P=0.5016 | P=0.8971 | P=0.0138* | 0.306 |
| Right amygdala | F (3, 22) = 4.452  P=0.0137 | P=0.9993 | P=>0.9999 | P=0.015* | P=0.9997 | P=0.2227 | P=0.0228* | 0.375 |
| Left amygdala | F (3, 15) = 0.4104  P=0.7479 | P=0.9982 | P=0.9341 | P=0.9968 | P=0.8952 | P=>0.999 | P=0.7488 | 0.075 |

*p- values from AD analysis in the offspring of mice according to prenatal diet exposure.*
